# Efficacy and safety evaluation of adalimumab for psoriatic arthritis: A meta-analysis

**DOI:** 10.1097/MD.0000000000050295

**Published:** 2026-08-21

**Authors:** Zhiyue Cai, Le Yan, Yue Zhang

**Affiliations:** a The Second School of Clinical Medicine, Zhejiang Chinese Medical University, Hangzhou, Zhejiang, China; b School of Medical and Life Sciences, Chengdu University of Traditional Chinese Medicine, Chengdu, China; c School of Materials Science and Engineering, China University of Petroleum (East China), Qingdao, Shandong, China.

**Keywords:** adalimumab, adverse events, efficacy, meta-analysis, psoriatic arthritis

## Abstract

**Background:**

This study aims to conduct a systematic evaluation of the efficacy and safety of adalimumab (ADA) in treating psoriatic arthritis (PsA), providing evidence-based reference data for clinical medicine.

**Methods:**

A systematic search was conducted in PubMed, Embase, Web of Science, the Cochrane Library, as well as Chinese databases, including China National Knowledge Infrastructure, WanFang Data, and VIP Chinese Scientific Journals Database, to identify relevant randomized controlled trials of ADA for PsA. The final search was updated on June 10, 2026. Data were analyzed using Stata 15.0.

**Results:**

This meta-analysis involved 17 studies, including 1 phase II clinical trial and 11 phase III clinical trials, covering 5655 patients. According to the meta-analysis, ADA demonstrated significant improvements in ACR20 (risk ratio [RR]: 2.27, 95% confidence interval [CI]: 1.83–2.83), ACR50 (RR: 3.92, 95% CI: 2.98–5.15), and ACR70 (RR: 5.75, 95% CI: 4.47–7.39) among PsA patients compared with the control group. In addition, it also improved PASI75 (RR: 7.07, 95% CI: 4.36–11.46), PASI90 (RR: 4.27, 95% CI: 1.64–11.14), and PASI100 (RR: 8.23, 95% CI: 3.89–17.40). Furthermore, ADA was associated with improvements in other relevant indicators, including PsA response criteria (RR: 2.45, 95% CI: 2.01–2.99), complete resolution of enthesitis (RR: 1.46, 95% CI: 1.22–1.74), and minimal disease activity (RR: 3.10, 95% CI: 2.58–3.73). The study also performed subgroup analyses for outcomes at different stages and dosing intervals. The results showed that there was no significant increase in the overall risk of adverse events (AEs; RR = 1.04) or serious AEs (RR = 1.13), and there was no notable increase in discontinuations due to AEs (RR = 1.46). In the context of specific AEs, ADA was associated with 11 types of AEs but showed almost no impact on the other 6 AEs.

**Conclusion:**

ADA demonstrated beneficial efficacy in treating PsA, as evidenced by improvements in relevant indicators. Meanwhile, ADA demonstrated overall clinical efficacy; however, it was associated with a significantly increased risk of elevated liver enzymes (alanine aminotransferase and aspartate aminotransferase), suggesting a potential signal of hepatotoxicity. While no increase in overall or serious AEs was observed, liver function monitoring may be warranted during treatment.

## 1. Introduction

Psoriatic arthritis (PsA), a chronic inflammatory joint condition associated with psoriasis,^[1]^ is characterized by high heterogeneity and complex clinical manifestations.^[2]^ The pathogenesis of PsA involves multiple factors, including genetics, environment, and immunity.^[3–5]^ The aberrant activation of the immune system leads to increased levels of pro-inflammatory cytokines, including tumor necrosis factor-alpha (TNF-α), interleukin-23 (IL-23), and interleukin-17 (IL-17). TNF-α induces the overexpression of matrix metalloproteinase and receptor activator of nuclear factor kappa-B ligand in synovial fibroblasts and chondrocytes,^[6,7]^ thereby promoting cartilage degradation and bone loss,^[8,9]^ which ultimately results in the characteristic irreversible joint structural damage observed in PsA.^[5]^ Epidemiological studies reveal that the cumulative incidence of PsA among patients with psoriasis ranges from approximately 6% to 41%, with the risk increasing alongside the duration of the disease, particularly peaking between the ages of 30 and 60 years.^[10]^ Therefore, there is an urgent need for biological agents that target key immune pathways in order to improve outcomes.

The basic medications for PsA are nonsteroidal anti-inflammatory drugs and disease-modifying antirheumatic drugs (DMARDs). In clinical practice, nonsteroidal anti-inflammatory drugs should only be used as an additional medication, as they alleviate symptoms of pain but do not affect the progression of the disease itself.^[11]^ Actually, the true corrective treatment for PsA began with the application of DMARDs,^[12]^ including conventional synthetic DMARDs (csDMARDs), biologic DMARDs (bDMARDs), and targeted synthetic DMARDs. Although csDMARDs have been shown to be somewhat effective in alleviating the symptoms of peripheral arthritis in patients with PsA, there is still considerable debate about their use in clinical practice.^[13]^ Therefore, over the past 6 years and even longer, researchers have been dedicated to addressing this core issue and have achieved breakthrough progress. According to several recent authoritative guidelines,^[11]^ if patients with active peripheral PsA have failed to tolerate or respond to 1 or more csDMARDs, this signals the entry of PsA treatment into an era of targeted precision therapy based on key pathogenic cytokines or signaling pathways.^[14]^ Unlike csDMARDs, bDMARDs target key inflammatory mediators precisely, thereby controlling joint, skin, entheseal, and axial pathologies simultaneously.^[15,16]^ TNF-α inhibitors (including adalimumab (ADA), infliximab, etanercept, golimumab, certolizumab pegol, and others) are the first bDMARDs approved for PsA and remain one of the most commonly used classes to date.^[17,18]^

ADA is a fully human monoclonal antibody that targets TNF-α.^[19]^ Its therapeutic mechanism involves binding specifically to TNF-α, thereby blocking its interaction with the p55 and p75 receptors and inhibiting the inflammatory signaling pathways mediated by TNF-α. Furthermore, in the presence of complement, it can lyse cells that express tumor necrosis factor on their surfaces, thereby reducing inflammatory responses and immune-mediated damage.^[20]^ ADA has received approval from both the Food and Drug Administration and European Medicines Agency and is currently used to treat a range of chronic immune-mediated inflammatory diseases, including rheumatoid arthritis,^[21]^ ankylosing spondylitis,^[22]^ PsA,^[11]^ moderate-to-severe chronic plaque psoriasis,^[23]^ moderately to severely active Crohn disease,^[24]^ ulcerative colitis,^[25]^ noninfectious uveitis,^[26]^ and juvenile idiopathic arthritis.^[27]^ Long-term follow-up data spanning over a decade demonstrate that ADA maintains its therapeutic efficacy, achieving a drug retention rate of between 72% and 82%. It has a consistently favorable safety profile and offers sustained benefits for patients.^[28]^

Although similar topics have been addressed in previous studies,^[29]^ this meta-analysis provided significant new insights by systematically evaluating efficacy outcomes, including ACR20, ACR50, ACR70, PASI75, PASI90, and PASI100, alongside other relevant measures. It also comprehensively documented the incidence of adverse reactions. This study aimed to address PsA – a disabling articular manifestation of psoriasis – by systematically investigating the therapeutic role of ADA in patients with PsA and evaluating its efficacy and incidence of adverse reactions.

## 2. Methods and data

The study protocol was registered with International Prospective Register of Systematic Reviews (CRD420251145343) and conducted in accordance with the Preferred Reporting Items for Systematic Reviews and Meta-Analyses Protocols guidelines.^[30]^ This article is based on previously conducted studies and does not involve any new studies of human or animal subjects performed by any of the authors.

### 2.1. Search strategy

This study searched 4 databases, including PubMed, Web of Science, Embase, and the Cochrane Library, as well as major Chinese databases (China National Knowledge Infrastructure, WanFang Data, and VIP Chinese Scientific Journals Database), with the retrieval of relevant studies commencing on June 10, 2026. Given the significant variation in authors’ preferred nomenclature for pharmaceutical agents, this study employed a comprehensive search strategy that integrated both free-text terms and medical subject headings. In this study, search terms included (“adalimumab” OR “Humira” OR “anti-TNF”) AND (“psoriatic arthritis” OR “PsA” OR “psoriatic arthropathy”) AND (randomized controlled trial OR RCT). Additionally, this meta-analysis performed a manual search of the relevant literature.

### 2.2. Inclusion and exclusion criteria

Inclusion criteria: Patients who met the diagnostic criteria for PsA, were aged >18 years, and received ADA were enrolled in the experimental group, while those who took a placebo for PsA were included in the control group. The main outcome indicators were ACR20, ACR50, ACR70, PASI75, PASI90, PASI100, other relevant clinical indicators, and adverse events (AEs). Randomized controlled trials (RCTs) that fulfilled the above-described criteria were considered for inclusion.

Exclusion criteria: Articles that were not published in English or were non-phase II or III clinical trials were excluded; studies were excluded if they incorporated inappropriate subgroup analyses or consisted of single-arm investigations lacking a comparative control group; research data could not be extracted; and if repeated studies were published, the latest article was used.

### 2.3. Data extraction

To ensure that the data were entered objectively and accurately, 2 researchers independently extracted the baseline data for each study, while disagreements were resolved by a third investigator. If no outcome data were available, the corresponding authors were contacted for further information. The following baseline information was extracted: authors, publication period, ClinicalTrials.gov number, the phase of the clinical trials, the medication regimen of the ADA group and the placebo group, trial enrollment size, primary outcome measures such as ACR20, ACR50, ACR70, PASI75, PASI90, PASI100, other relevant measures, and AEs. If the outcomes of various studies involved different phases or dosing intervals, this research performed subgroup analyses to account for these variations.

### 2.4. Quality appraisal

Two investigators independently assessed the risk of bias using version 2 of the Cochrane risk-of-bias tool for randomized trials (RoB 2, Cochrane, London, UK),^[31]^ including the following 7 aspects: random sequence generation, allocation concealment, blinding of patients and personnel, blinding of outcome assessors, outcome data integrity, selective reporting, and others. The risk of bias was rated as low, high, or unclear for each major study. Any disputes in the judgments were discussed.

### 2.5. Statistical analysis

The efficacy outcomes, including ACR20, ACR50, ACR70, PASI75, PASI90, PASI100, as well as other relevant endpoints, were assessed using risk ratios (RRs) with 95% confidence intervals (CIs) to compare the ADA group with the placebo group, while the AEs were also evaluated using RRs with 95% CIs. For rare AEs, studies with 0 events in 1 arm were analyzed using a continuity correction of 0.5. Studies with 0 events in both arms were excluded from the quantitative synthesis. Sensitivity analyses were performed using alternative statistical models to assess the robustness of the pooled estimate. To assess heterogeneity, *I*^2^ and *Q* tests were conducted. If the *I*^2^ statistic exceeded 50%, a random-effects model was adopted. Otherwise, if it was below 50%, a fixed-effects model was used. A sensitivity analysis was performed in this study using a step-by-step exclusion of individual studies, given that the *I*^2^ statistic exceeded 50%. To assess potential publication bias, this study employed funnel plots and Egger’s test. *P* > .05 indicated that no publication bias was detected. The funnel plot was asymmetric. Data analysis was performed using Stata software, version 15.0 (StataCorp LLC, College Station).

## 3. Results

### 3.1. Literature screening and results

A total of 2845 records were identified through database searching, including PubMed, Embase, Web of Science, the Cochrane Library, China National Knowledge Infrastructure, WanFang Data, and VIP Chinese Scientific Journals Database, and 8 additional records were identified through other sources. After removing duplicates, 1195 records remained for screening. Following title and abstract screening, 322 records were assessed for eligibility, of which 256 were excluded. A total of 66 full-text articles were evaluated for eligibility, and 49 were excluded for the following reasons: not phase II or III RCTs (n = 23), inappropriate treatment regimens (n = 14), insufficient data for extraction (n = 9), and duplicate or updated publications (n = 3). Ultimately, 17 studies^[32–48]^ were included in the qualitative and quantitative synthesis. The detailed search and screening processes were illustrated in Figure S1, Supplemental Digital Content 1.

### 3.2. Research-related characteristics

ADA, which is a biologic agent, belongs to the category of TNF-α monoclonal antibodies. Among these 17 included studies, 7 studies evaluated ADA versus placebo. In addition, 2 studies evaluated ADA, ixekizumab, and placebo, while another 2 investigations involved ADA and tofacitinib, along with placebo. Besides, 2 trials utilized ADA, upadacitinib, and placebo in their design, and a further 2 studies incorporated ADA, bimekizumab, and a placebo group. Finally, 2 studies made a comparison of ADA, ABT-122 (a bispecific antibody developed by AbbVie that simultaneously targets TNF-α and IL-17A), and a placebo group, as well as ADA, methotrexate (MTX), and a placebo group, respectively. Regarding the outcome measures, 8 studies utilized ACR20, ACR50, and ACR70 as their endpoints, while PASI75, PASI90, and PASI100 outcome indicators were evaluated in 9 studies. Because the included studies had particular characteristics, there were 12 outcomes evaluated over a 0 to 12-week period, while 8 endpoints focused specifically on the 0 to 16-week period. Additionally, 9 measurements were recorded from baseline to 24 weeks. The included research features are detailed in Table S1, Supplemental Digital Content 2.

### 3.3. Quality appraisal

All of the included trials were carried out and strictly followed the study design, and as a result, exhibited a low risk of bias across the following 3 aspects: complete blinding of both patients and study personnel was implemented in all studies, outcome data were presented with full completeness, and no instances of selective reporting were observed. The majority of biases were primarily due to uncertainty surrounding the generation of a proper random sequence and the concealment of adequate allocation, both of which were unclear in more than half of the studies, as well as insufficient blinding of outcome assessors. The risk of bias assessment for the included studies was shown in Figures S2 and S3, Supplemental Digital Content 3.

### 3.4. Meta-analysis results

#### 3.4.1. ACR20

**Figure 1.**
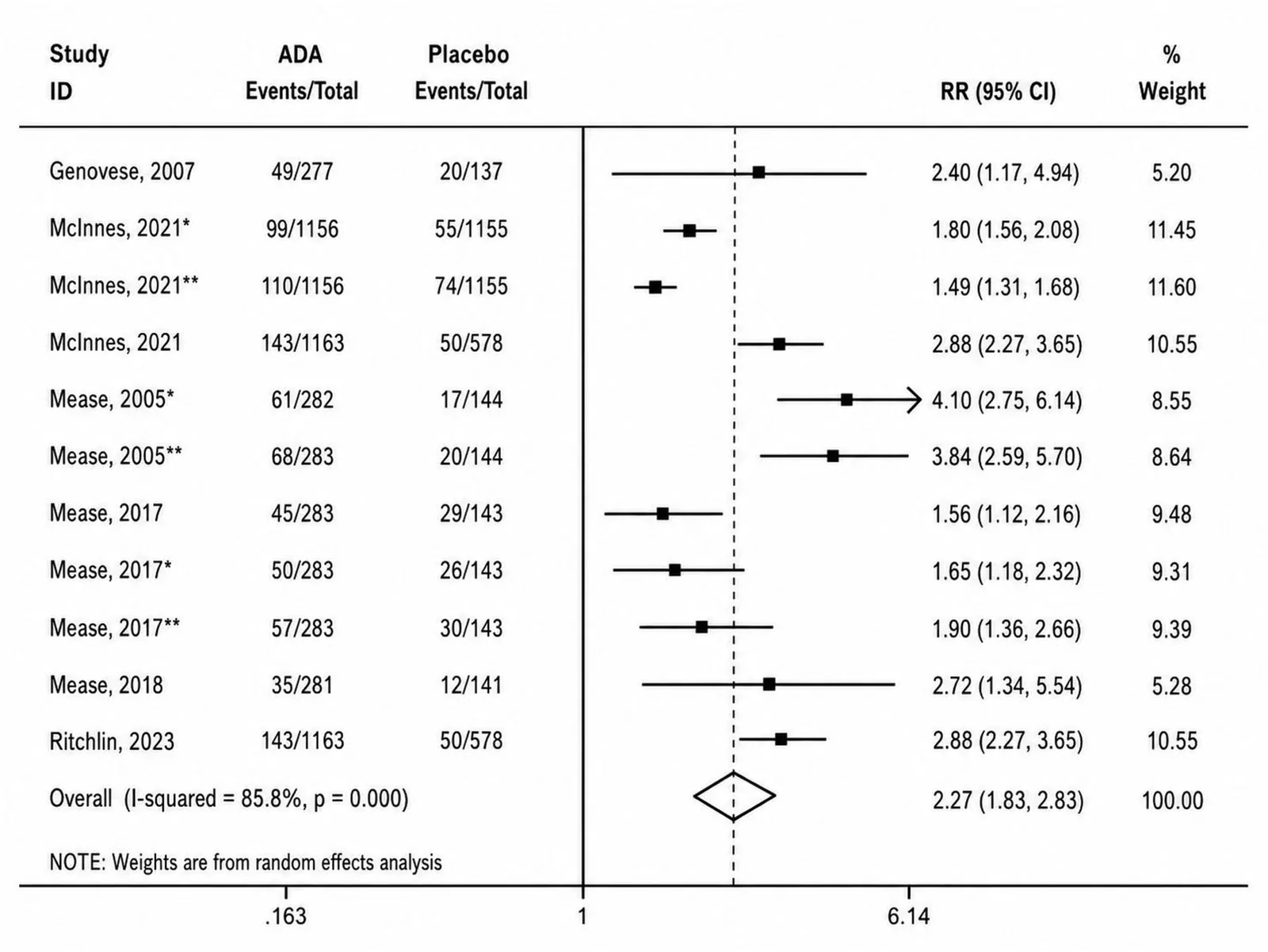
Forest plot comparing the differences in the ACR20 indicator between the use of ADA and the use of placebo. ACR = American College of Rheumatology response criteria, ADA = adalimumab, CI = confidence interval, RR = risk ratio.

The indicator of ACR20 was specifically reported in 8 studies. The results demonstrated that, when compared with the ADA group, the placebo group exhibited a significantly elevated relative risk (RR: 2.27, 95% CI: 1.83–2.83). As indicated by the heterogeneity test (*I*^2^ = 85.8%, *P* < .001), significant heterogeneity was observed across the included studies; therefore, a random-effects model was applied for the analysis, as shown in Figure 1. The sensitivity analysis, performed by sequentially excluding each study, showed that the pooled RR ranged from 2.18 to 2.41, and the direction of effect remained unchanged. No single study significantly altered the overall effect size, indicating that the results were robust and stable; see Figure S4, Supplemental Digital Content 5. The Egger’s value for ACR20 was 0.063, indicating no evidence of publication bias. Because of the asymmetry of the funnel plot, as shown in Figure S5, Supplemental Digital Content 6, a trimming method was employed. The analysis results suggested that ADA had a beneficial effect on ACR20. The substantial heterogeneity observed in ACR20 (*I*^2^ = 85.8%) may be attributed to differences in study-level characteristics, including variations in ADA dosing schedules, treatment duration, baseline disease severity, and concomitant therapies such as MTX or other DMARDs. Despite this heterogeneity, subgroup and sensitivity analyses demonstrated consistent effect directions across studies.

#### 3.4.2. ACR50

**Figure 2.**
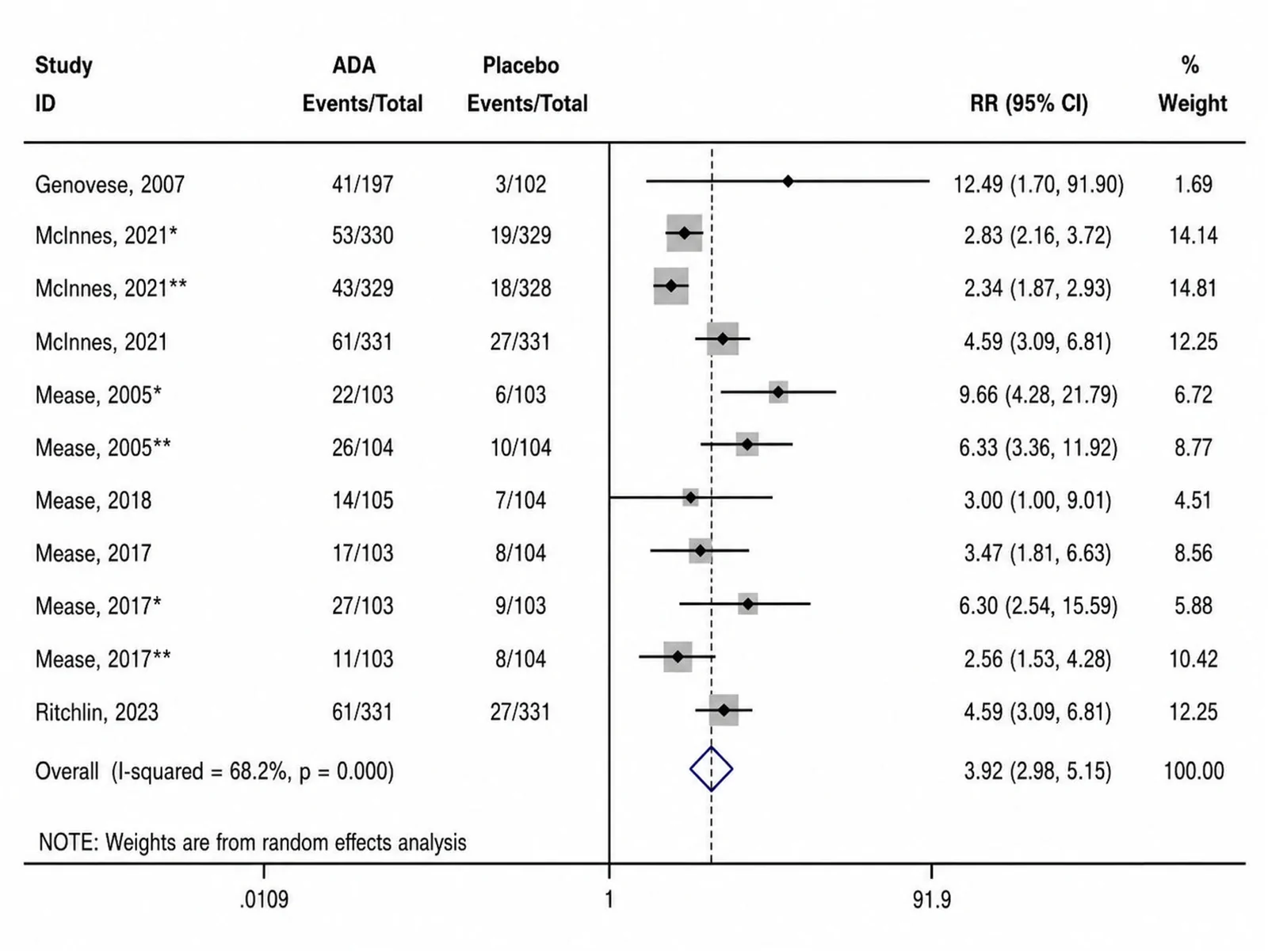
Forest plot comparing the differences in the ACR50 indicator between the use of ADA and the use of placebo. ACR = American College of Rheumatology response criteria, ADA = adalimumab, CI = confidence interval, RR = risk ratio.

Eight distinct studies documented the ACR50 outcome, which likewise revealed an increased RR in the placebo group relative to the ADA group (RR: 3.92, 95% CI: 2.98–5.15). As indicated by the heterogeneity test (*I*^2^ = 68.2%, *P* < .001), significant heterogeneity was observed across the included studies; therefore, a random-effects model was applied for the analysis, as shown in Figure 2. The pooled RR ranged from 3.76 to 4.08 after omitting individual studies one by one. The overall effect direction remained consistent, suggesting that the findings were not driven by any single study; see Figure S6, Supplemental Digital Content 7. Egger’s test suggested potential publication bias (*P* = .018), as shown in Figure S7, Supplemental Digital Content 8. The analysis results suggested that ADA had a beneficial effect on ACR50.

#### 3.4.3. ACR70

**Figure 3.**
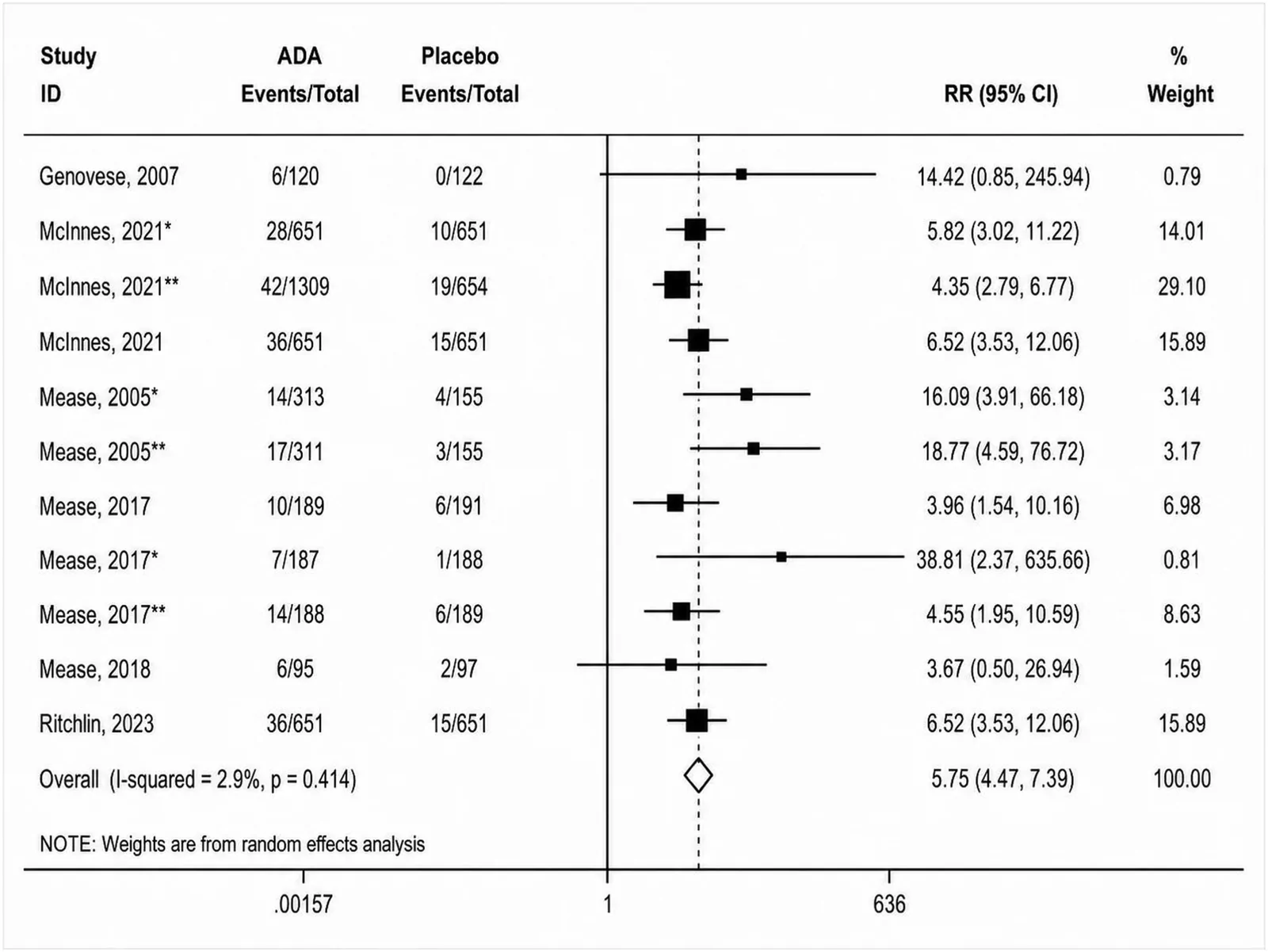
Forest plot comparing the differences in the ACR70 indicator between the use of ADA and the use of placebo. ACR = American College of Rheumatology response criteria, ADA = adalimumab, CI = confidence interval, RR = risk ratio.

Eight studies evaluated the endpoint of ACR70 and found higher RR values in the placebo group than in the ADA group (RR: 5.75, 95% CI: 4.47–7.39). As indicated by the heterogeneity test (*I*^2^ = 2.9%, *P* = .414), low heterogeneity was observed across the included studies, as shown in Figure 3. The Egger’s value for ACR70 was 0.046, indicating no evidence of publication bias. Because of the asymmetry in the funnel plot, this study employed a trimming method (*P* = .243). The results indicated that publication bias did not compromise the reliability of the conclusions, as shown in Figure S8, Supplemental Digital Content 9. The analysis results suggested that ADA had a beneficial effect on ACR70.

#### 3.4.4. PASI75

**Figure 4.**
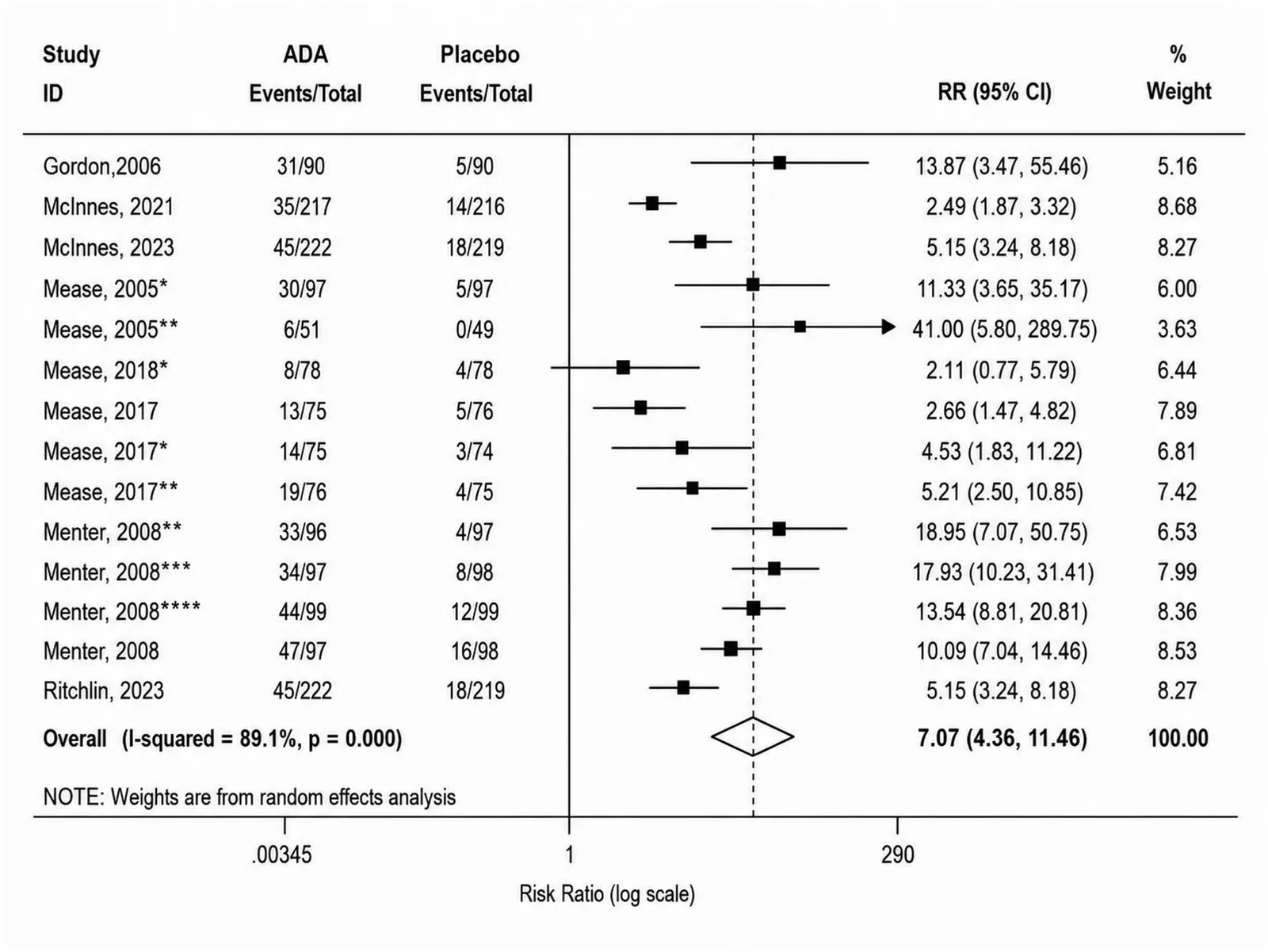
Forest plot comparing the differences in the PASI75 indicator between the use of ADA and the use of placebo. ADA = adalimumab, CI = confidence interval, PASI = Psoriasis Area and Severity Index, RR = risk ratio.

The indicator of PASI75 was specifically reported in 9 studies. The results demonstrated that, when compared with the ADA group, the placebo group exhibited a significantly elevated relative risk (RR: 7.07, 95% CI: 4.36–11.46). As indicated by the heterogeneity test (*I*^2^ = 89.1%, *P* < .001), significant heterogeneity was observed across the included studies; therefore, a random-effects model was applied for the analysis, as shown in Figure 4. Sensitivity analysis showed that the pooled RR varied between 6.82 and 7.29, with no reversal of effect direction. The results were therefore considered stable; see Figure S9, Supplemental Digital Content 10. The Egger’s value for PASI75 was 0.268, indicating no evidence of publication bias, as shown in Figure S10, Supplemental Digital Content 11. The analysis results suggested that ADA had a beneficial effect on PASI75. The substantial heterogeneity observed in PASI75 (*I*^2^ = 89.1%) may be attributed to differences in study-level characteristics, including variations in ADA dosing schedules, treatment duration, baseline disease severity, and concomitant therapies such as MTX or other DMARDs. Despite this heterogeneity, subgroup and sensitivity analyses demonstrated consistent effect directions across studies.

#### 3.4.5. PASI90

**Figure 5.**
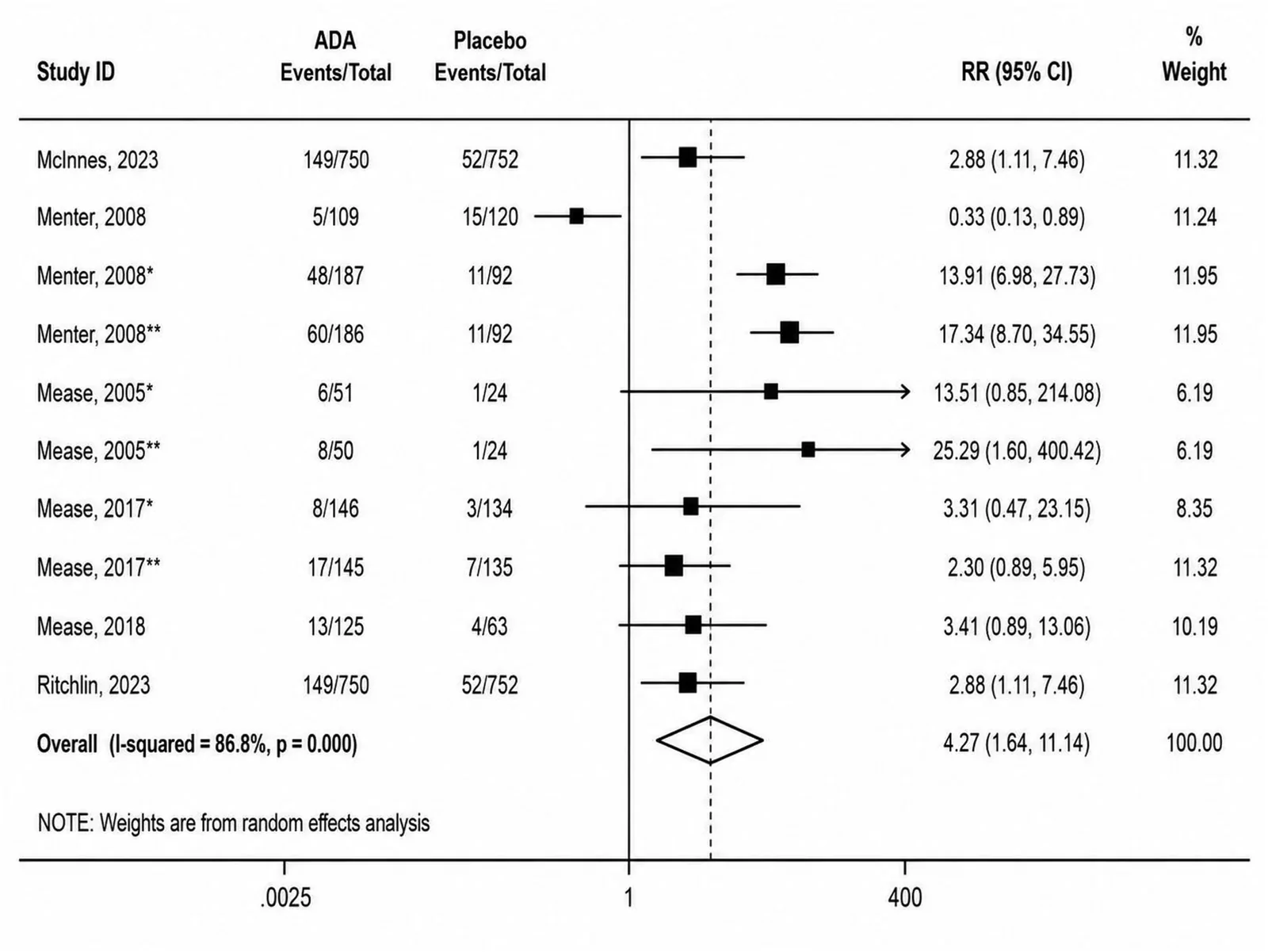
Forest plot comparing the differences in the PASI90 indicator between the use of ADA and the use of placebo. ADA = adalimumab, CI = confidence interval, PASI = Psoriasis Area and Severity Index, RR = risk ratio.

Six distinct studies documented the PASI90 outcome, which likewise revealed an increased RR in the placebo group relative to the ADA group (RR: 4.27, 95% CI: 1.64–11.14). As indicated by the heterogeneity test (*I*^2^ = 86.8%, *P* < .001), significant heterogeneity was observed across the included studies; therefore, a random-effects model was applied for the analysis, as shown in Figure 5. The leave-one-out sensitivity analysis showed minimal variation in pooled effect sizes, and no individual study materially influenced the overall results, confirming the robustness of the findings; see Figure S11, Supplemental Digital Content 12. The Egger’s value for PASI90 was 0.602, indicating the presence of publication bias. Because of the asymmetry of the funnel plot, as shown in Figure S12, Supplemental Digital Content 13, a trimming method was employed. The analysis results suggested that ADA had a beneficial effect on PASI90.

#### 3.4.6. PASI100

**Figure 6.**
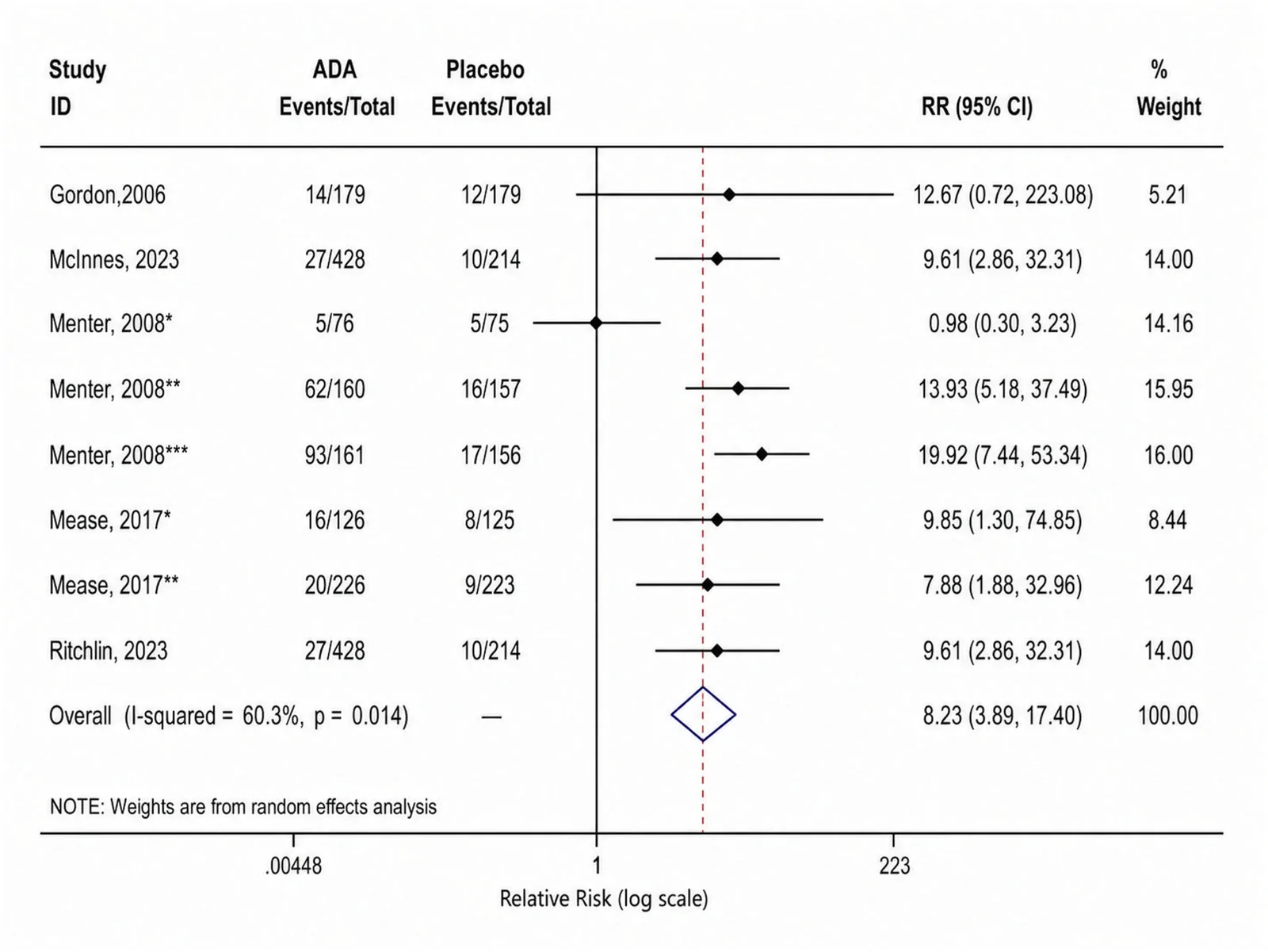
Forest plot comparing the differences in the PASI100 indicator between the use of ADA and the use of placebo. ADA = adalimumab, CI = confidence interval, PASI = Psoriasis Area and Severity Index, RR = risk ratio.

Five studies evaluated the endpoint of PASI100 and also found higher RR values in the placebo group than in the ADA group (RR: 8.23, 95% CI: 3.89–17.40). As indicated by the heterogeneity test (*I*^2^ = 60.3%, *P* = .014), significant heterogeneity was observed across the included studies; therefore, a random-effects model was applied for the analysis, as shown in Figure 6. The leave-one-out sensitivity analysis showed minimal variation in pooled effect sizes, and no individual study materially influenced the overall results, confirming the robustness of the findings (see Fig. S13, Supplemental Digital Content 14). The Egger’s value for PASI100 was 0.792, indicating no presence of publication bias. Because of the asymmetry of the funnel plot, as shown in Figure S14, Supplemental Digital Content 15, a trimming method was employed. The analysis results suggested that ADA had a beneficial effect on PASI100, based on the evaluation of the data.

#### 3.4.7. Other relevant indicators

**Figure 7.**
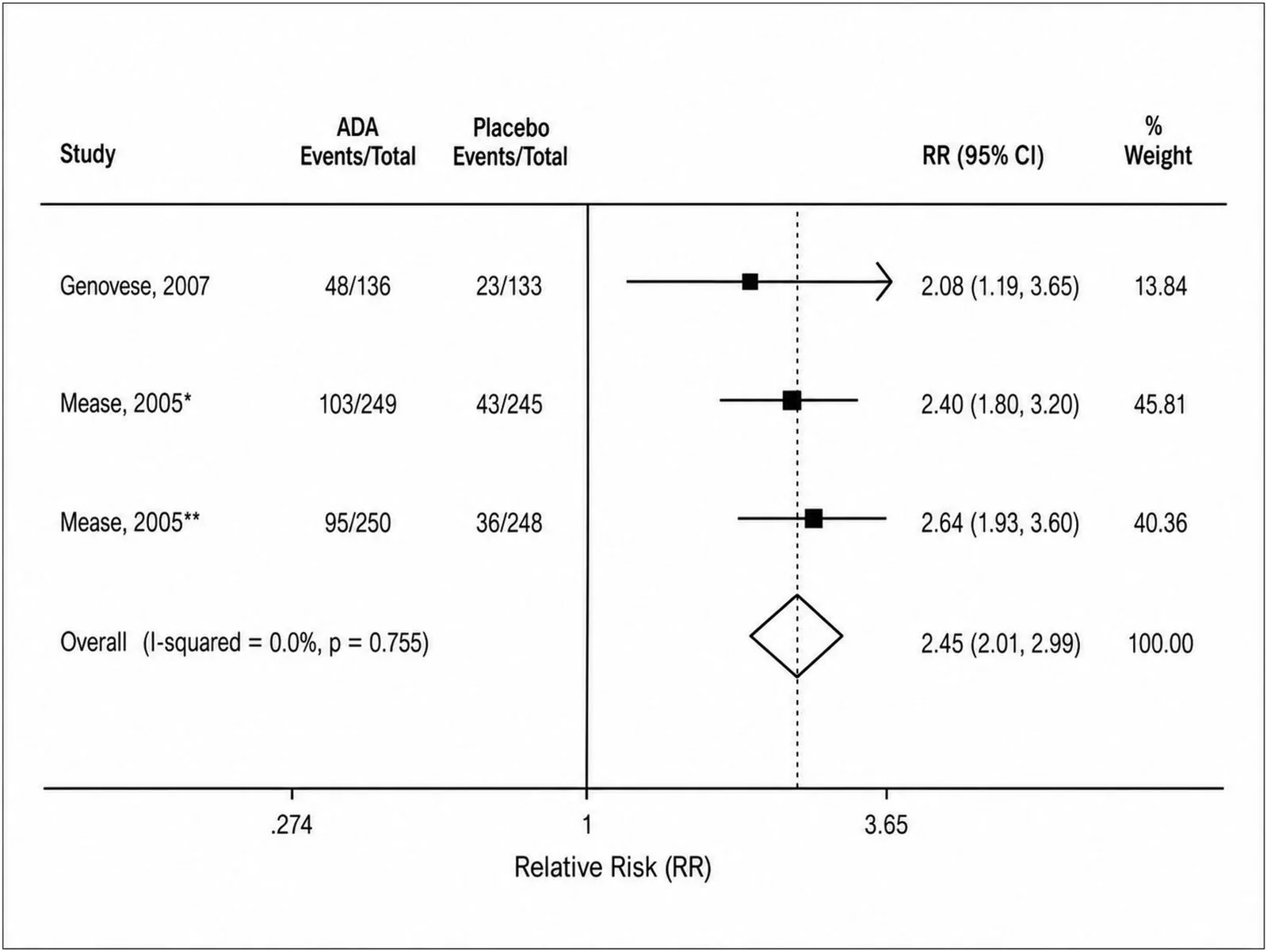
Forest plot comparing the differences in PsARC between the use of ADA and the use of placebo. ADA = adalimumab, CI = confidence interval, PsARC = Psoriatic Arthritis Response Criteria, RR = risk ratio.

The indicator of Psoriatic Arthritis Response Criteria (PsARC) was specifically reported in 2 studies. The results demonstrated that, when compared with the ADA group, the placebo group exhibited a significantly elevated relative risk (RR: 2.45, 95% CI: 2.01–2.99). The heterogeneity test (*I*^2^ = 0.0%, *P* = .755) indicated no heterogeneity among studies; thus, a fixed-effects model was employed for analysis, as shown in Figure 7. The analysis results suggested that ADA had a beneficial effect on PsARC, based on the evaluation of the data.

**Figure 8.**
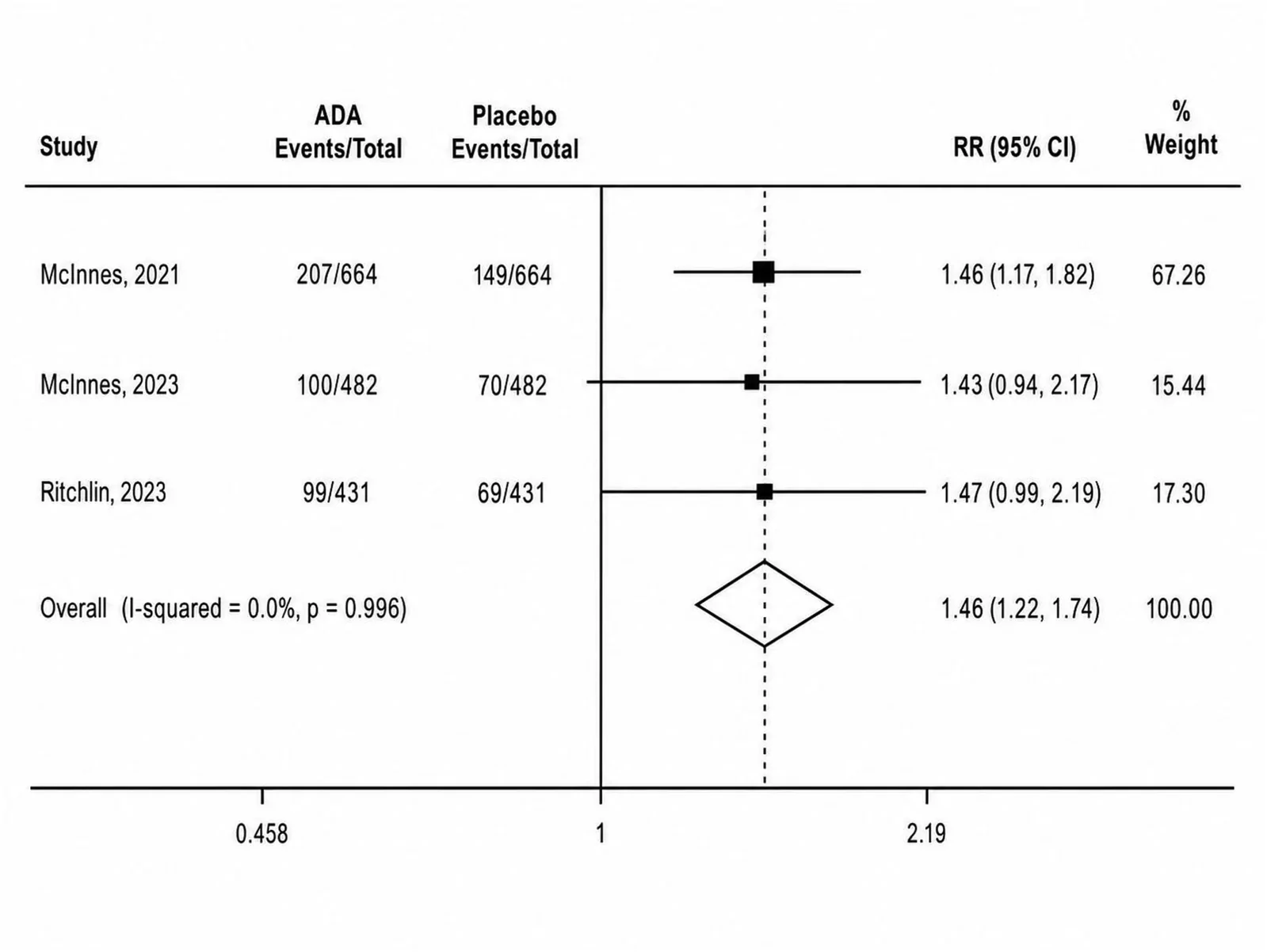
Forest plot comparing the differences in complete resolution of enthesitis between the use of ADA and the use of placebo. ADA = adalimumab, CI = confidence interval, RR = risk ratio.

Three distinct studies documented the complete resolution of enthesitis outcome, which likewise revealed an increased RR in the placebo group relative to the ADA group (RR: 1.46, 95% CI: 1.22–1.74). The heterogeneity test (*I*^2^ = 0.0%, *P* = .996) indicated no heterogeneity among studies; thus, a fixed-effects model was employed for analysis, as shown in Figure 8. The analysis results suggested that ADA had a beneficial effect on the complete resolution of enthesitis, based on the evaluation of the data.

**Figure 9.**
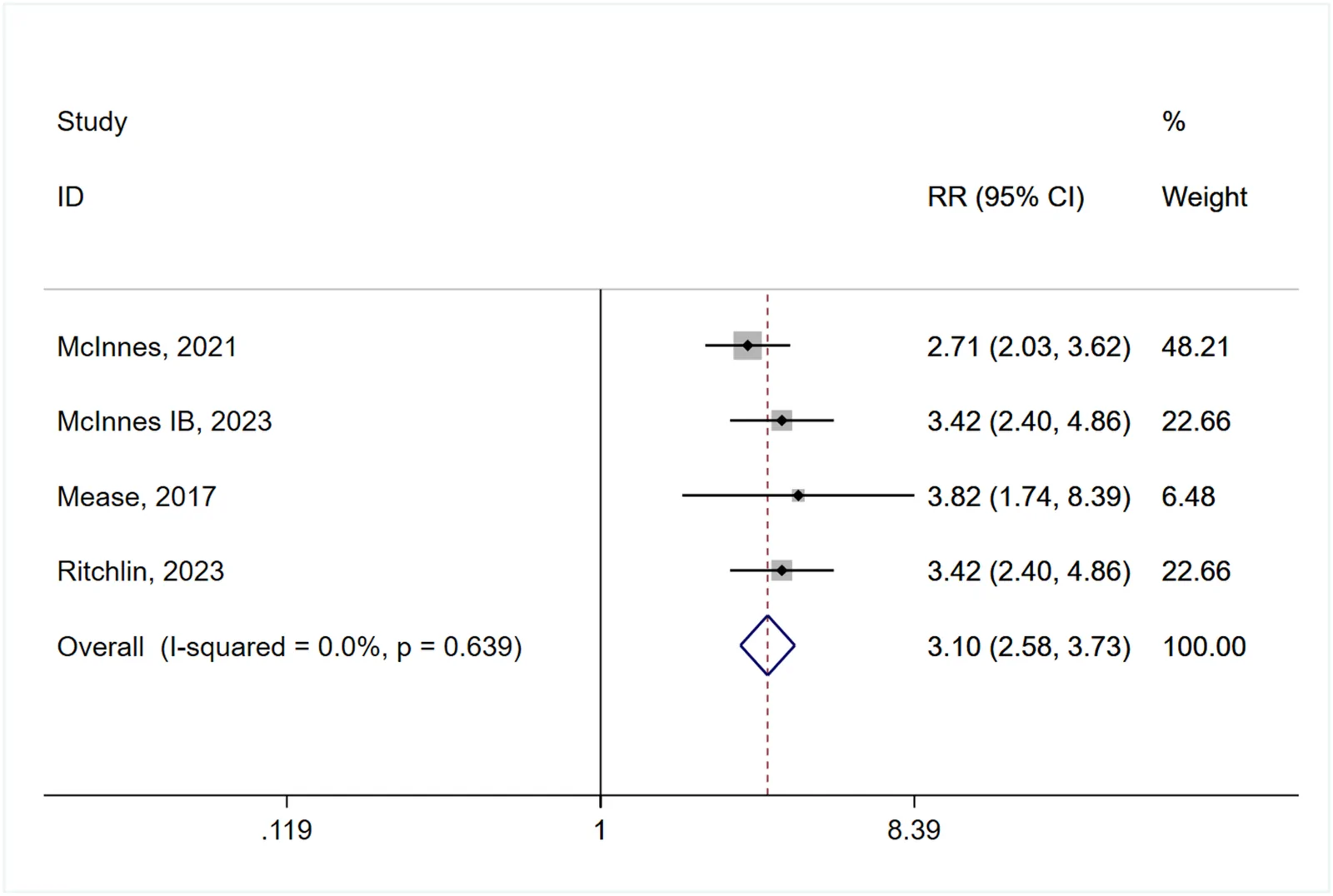
Forest plot comparing the differences in MDA between the use of ADA and the use of placebo. ADA = adalimumab, CI = confidence interval, MDA = minimal disease activity, RR = risk ratio.

Four studies evaluated the endpoint of minimal disease activity (MDA) and also found higher RR values in the placebo group than in the ADA group (RR: 3.10, 95% CI: 2.58–3.73). The heterogeneity test (*I*^2^ = 0.0%, *P* = .639) indicated no heterogeneity among studies; thus, a fixed-effects model was employed for analysis, as shown in Figure 9. The analysis results suggested that ADA had a beneficial effect on MDA, based on the evaluation of the data.

#### 3.4.8. Adverse events

The results showed that there was no significant increase in the overall risk of AEs (RR = 1.04) or serious AEs (RR = 1.13), and there was no notable increase in discontinuations due to AEs (RR = 1.46). In the context of specific AEs, ADA was associated with 11 types of AEs, including opportunistic infections (RR = 6.00), elevated alanine aminotransferase (ALT) levels (RR = 4.09), elevated aspartate aminotransferase (AST) levels (RR = 5.41), neutropenia (RR = 3.62), depression (RR = 3.07), injection-site reactions (RR = 1.92), serious infections (RR = 1.69), urinary tract infections (RR = 1.45), back pain (RR = 1.32), nausea (RR = 1.23), and death (RR = 1.01). ADA, however, showed almost no impact on the other 6 AEs, including nasopharyngitis (RR = 0.99), diarrhea (RR = 0.90), headache (RR = 0.93), PsA aggravated (RR = 0.55), psoriasis aggravated (RR = 0.28), and hypertension (RR = 0.98). The detailed results were illustrated in Table 1.

**Table 1:** A meta-analysis summary table of AEs caused by the use of adalimumab.

Adverse event	Study	Heterogeneity		RR (95% CI)	*P*
		*P*	*I*^2^ (%)		
Any AE	9	.083	42.6	1.04 (0.94–1.16)	.411
Any serious AE	11	.756	0.0	1.13 (0.73–1.74)	.591
Discontinuation due to AE	10	.830	0.0	1.46 (0.93–2.29)	.097
Neutropenia	4	.656	0.0	3.62 (1.01–13.04)	.049
Nausea	4	.583	0.0	1.23 (0.47–3.23)	.674
Diarrhea	5	.382	4.3	0.90 (0.49–1.67)	.747
Headache	6	.378	6.0	0.93 (0.59–1.47)	.771
PsA aggravated	3	.129	51.2	0.55 (0.13–2.27)	.406
Psoriasis aggravated	2	.770	0.0	0.28 (0.11–0.75)	.011
Injection-site reaction	7	.418	0.7	1.92 (1.21–3.06)	.006
Hypertension	3	.465	0.0	0.98 (0.52–1.87)	.954
Back pain	2	.092	64.7	1.32 (0.06–29.21)	.861
Depression	2	.983	0.0	3.07 (0.33–28.78)	.326
Urinary tract infection	3	.975	0.0	1.45 (0.54–3.92)	.465
Death	2	.333	0.0	1.01 (0.11–9.52)	.991
Nasopharyngitis	6	.790	0.0	0.99 (0.72–1.36)	.944
Opportunistic	2	1.000	0.0	6.00 (0.63–57.42)	.120
AST increased	3	.767	0.0	5.41 (1.45–20.22)	.012
ALT increased	4	.169	40.4	4.09 (1.05–15.85)	.042
Serious infection	4	.400	0.0	1.69 (0.54–5.34)	.371

AE = adverse event, ALT = alanine aminotransferase, AST = aspartate aminotransferase, CI = confidence interval, PsA = psoriatic arthritis, RR = risk ratio.

### 3.5. Subgroup analysis

The study also performed subgroup analyses for outcomes at different stages and dosing intervals.

#### 3.5.1. ACR20

Based on subgroup analysis results for the ACR20 endpoint, “every 2 weeks” (RR: 2.45, 95% CI: 1.75–3.42, *P* < .001) and “every 2 weeks” (RR: 2.14, 95% CI: 1.64–2.80, *P* = .002) showed no significant difference in intervention effects. Results were shown in Figure S15, Supplemental Digital Content 16. Stratified by dosing phase, “0 to 12 weeks” (RR: 2.33, 95% CI: 1.83–2.97, *P* < .001) and “12 to 24 weeks” (RR: 2.16, 95% CI: 1.26–3.70, *P* < .001) also showed no significant difference in intervention effects. Results were shown in Figure S16, Supplemental Digital Content 17.

#### 3.5.2. ACR50

Based on subgroup analysis results for the ACR50 endpoint, “every 2 weeks ” (RR: 3.99, 95% CI: 2.56–6.21, *P* = .001) and “every 2 weeks” (RR: 4.03, 95% CI: 3.12–5.20, *P* = .291) showed no significant difference in intervention effects. Results are shown in Figure S17, Supplemental Digital Content 18. When stratified by treatment phase, the RR value was higher in the 0 to 12 weeks phase (RR: 4.32, 95% CI: 3.21–5.82, *P* = .048) compared with the 12 to 24 weeks phase (RR: 3.16, 95% CI: 1.85–5.41, *P* = .013). Results are shown in Figure S18, Supplemental Digital Content 19.

#### 3.5.3. ACR70

Based on the results of the ACR70 subgroup analysis, the RR value was higher in the every 2 weeks dosing regimen (RR: 6.51, 95% CI: 3.93–10.76, *P* = .206) compared with the every 2 weeks regimen (RR: 5.87, 95% CI: 4.12–8.37, *P* = .542). Results are shown in Figure S19, Supplemental Digital Content 20. When stratified by treatment phase, the RR value was higher in the 0 to 12 weeks phase (RR: 6.39, 95% CI: 4.64–8.81, *P* *=* .604) compared with the 12 to 24 weeks phase (RR: 5.64, 95% CI: 2.89–10.99, *P* = .135). Results are shown in Figure S20, Supplemental Digital Content 21.

#### 3.5.4. PASI75

Based on the results of the PASI75 subgroup analysis, the RR value was higher in the every 2 weeks dosing regimen (RR: 7.85, 95% CI: 2.67–23.11, *P* < .001) compared with the every 2 weeks regimen (RR: 4.51, 95% CI: 3.49–5.83, *P* = .432). Results are shown in Figure S21, Supplemental Digital Content 22. When stratified by treatment phase, the RR value was higher in the 12 to 24 weeks phase (RR: 12.44, 95% CI: 1.29–119.63, *P* = .028) compared with the 0 to 12 weeks phase (RR: 5.11, 95% CI: 2.95–8.83, *P* < .001). Results are shown in Figure S22, Supplemental Digital Content 23.

#### 3.5.5. PASI90

Based on the results of the PASI90 subgroup analysis, the RR value was higher in the every 2 weeks regimen (RR: 9.97, 95% CI: 4.34–22.86, *P* = .266) compared with the every 2 weeks dosing regimen (RR: 2.72, 95% CI: 1.60–4.60, *P* = .979). Results are shown in Figure S23, Supplemental Digital Content 24. When stratified by treatment phase, the RR value was higher in the 12 to 24 weeks phase (RR: 5.88, 95% CI: 0.39–89.88, *P* = .056) compared with the 0 to 12 weeks phase (RR: 4.88, 95% CI: 2.18–10.93, *P* *=* .019). Results are shown in Figure S24, Supplemental Digital Content 25.

#### 3.5.6. PASI100

Based on the results of the PASI100 subgroup analysis, the RR value was higher in the every 2 weeks dosing regimen (RR: 13.79, 95% CI: 5.41–35.16, *P* = .951) compared with the every 2 weeks regimen (RR: 9.20, 95% CI: 4.61–18.37, *P* = .996). Results are shown in Figure S25, Supplemental Digital Content 26. When stratified by treatment phase, the RR value was higher in the 0 to 12 weeks phase (RR: 11.19, 95% CI: 6.12–20.47, *P* = .985) compared with the 12 to 24 weeks phase (RR: 7.88, 95% CI: 1.88–32.96). Results are shown in Figure S26, Supplemental Digital Content 27.

## 4. Discussion

Although similar topics had been addressed in previous studies,^[29]^ this meta-analysis provides a more comprehensive evaluation of efficacy outcomes, including ACR20, ACR50, ACR70, PASI75, PASI90, and PASI100, as well as other clinically relevant measures. It also comprehensively documented the incidence of adverse reactions. The results demonstrated good efficacy and acceptable safety.

Multiple international studies have confirmed that the American College of Rheumatology (ACR) response criteria serve as a recognized endpoint in PsA clinical trials,^[49,50]^ encompassing a multidimensional framework that includes tender joint count (TJC), swollen joint count (SJC), pain visual analog scale (VAS), patient global assessment of disease activity (PtGA), and the Health Assessment Questionnaire-Disability Index, thus ensuring a comprehensive evaluation that balances objective inflammatory control with subjective patient experiences.^[51]^ For this reason, the ACR response criteria are widely used as the main endpoint in clinical studies around the world for evaluating the efficacy of biologics, including ADA.^[52]^ Furthermore, meeting the ACR response criteria is about more than just achieving a numerical target in clinical trials; it reflects and directly impacts patients’ personal experiences and quality of life.^[53]^ This research demonstrated that ADA significantly improved the efficacy of ACR20, ACR50, and ACR70 in patients with PsA. The findings of this study were also indirectly supported by a meta-analysis.^[54]^ Mechanistically, ADA binds with high affinity to both soluble TNF-α and transmembrane TNF-α, thereby blocking the p55 and p75 receptors. It also inhibits downstream inflammatory pathways.^[5]^ Ultimately, this leads to reduced synovial infiltration and decreased levels of interleukin-1, interleukin-6, and matrix metalloproteinases, alleviating synovitis, enthesitis, and skin lesions in patients with PsA.^[6,7]^

The Psoriasis Area and Severity Index (PASI) response criteria are not only regarded as the gold standard for measuring improvement in skin lesions,^[55]^ but also serve as a critical indicator for managing PsA and ultimately enhancing patients’ quality of life.^[56]^ The study found that patients with PsA using ADA could also achieve highly effective improvements in these types of indicators. The Adalimumab Effectiveness in Psoriatic Arthritis Trial, a phase III study in PsA, demonstrated that after 24 weeks of treatment with ADA, up to 59% of patients with moderate-to-severe skin psoriasis (affecting ≥ 3% of their body surface area) achieved a PASI75 improvement. This response rate was significantly higher than that observed in the placebo group.^[32]^ Thus, patients with PsA often experience skin and joint symptoms simultaneously – a clinical phenomenon with an underlying cause in the shared key inflammatory pathways, particularly the TNF-α and IL-23/IL-17 axes.^[57]^ By blocking TNF-α, ADA directly suppresses inflammation in both the synovium and the skin, while also indirectly decreasing the activity of the IL-23/IL-17 axis. This results in a broad and potent anti-inflammatory effect.^[58,59]^ It is precisely through this fundamental blockade of the core pathological pathways that ADA can improve joint swelling, tenderness, and structural damage while significantly increasing PASI75, PASI90, and PASI100 in skin lesions.

Furthermore, it was interesting to note that using ADA also led to significant improvements in other relevant indicators, making the findings highly informative and valuable. Designed to be efficient and comprehensive, PsARC is a core assessment tool for evaluating disease activity in PsA by integrating 4 key dimensions: TJC, SJC, patient global assessment (PtGA-VAS), and physician global assessment (VAS), enabling a holistic evaluation that typically takes <1 minute to complete.^[60]^ Moreover, achieving complete resolution of enthesitis is not only a key objective in PsA treatment but also a central indicator for preventing structural damage and improving quality of life.^[61]^ ADA demonstrates superior efficacy over placebo by precisely inhibiting TNF-α. This delays the onset and progression of enthesitis, which has significant clinical implications.^[20]^ MDA, as a comprehensive and practical set of criteria with strong prognostic relevance, encompasses multiple dimensions, including joint and skin involvement, physical function, pain, and patient-reported outcomes. It requires meeting specific targets such as TJC ≤ 1, SJC ≤ 1, PASI ≤ 1, or body surface area ≤ 3%, VAS pain ≤ 15 mm, PtGA ≤ 20 mm, Health Assessment Questionnaire-Disability Index ≤ 0.5, and the presence of no more than 1 enthesitis site, thereby providing a realistic reflection of the patient’s overall disease status and quality of life.^[62]^ Not only did ADA rapidly reduce inflammation in the joints and skin, but it also improved patients’ subjective symptoms and functional status. This slowed structural damage and significantly increased the rate at which the MDA was achieved.^[5,63]^ Although the above-mentioned indicators have received relatively little research attention to date, this paper argues that they represent a promising direction for future clinical research, given their critical role in evaluating patient symptoms, predicting disease outcomes, and informing treatment decisions. With the growing emphasis on patient-centered outcomes, composite measures such as PsARC, complete resolution of enthesitis, and MDA are set to become the “new standard endpoints,” replacing conventional indicators such as ACR and PASI. Consequently, they could provide essential criteria for evaluating drug therapies in the context of precision medicine and treat-to-target strategies.^[11]^

High heterogeneity was observed in several outcomes, particularly ACR20 and PASI75. This heterogeneity is likely explained by clinical and methodological differences across the included trials. First, treatment regimens varied among studies, including differences in dosing frequency (e.g., every 2 weeks vs fixed biweekly administration). Second, treatment duration ranged from short-term (0–12 weeks) to longer follow-up periods (12–24 weeks), which may influence response magnitude. Third, baseline disease severity and prior exposure to DMARDs were not uniform across studies. Finally, concomitant therapies were inconsistently reported and may have contributed to between-study variability. Nevertheless, the direction of treatment effect remained stable across sensitivity and subgroup analyses, suggesting that the overall conclusions are robust despite heterogeneity.

Subgroup analyses suggested that ADA may demonstrate differential effects across dosing regimens and treatment durations. However, these findings should be interpreted as exploratory and hypothesis-generating, as they were not adjusted for multiple comparisons and were based on limited numbers of studies. Therefore, no definitive conclusions regarding optimal dosing timing can be drawn from the present analysis.

Of clinical importance, this meta-analysis identified a significantly increased risk of elevated liver enzymes (ALT and AST) associated with ADA use. This finding suggests a potential signal of drug-induced liver injury, although the absolute incidence remains relatively low. The mechanism may be related to immune-mediated hepatotoxicity or idiosyncratic drug reactions. These results highlight the importance of routine liver function monitoring during treatment with TNF-α inhibitors, especially in patients with preexisting liver conditions or concomitant hepatotoxic medications.

### 4.1. Strengths and limitations

This study systematically evaluated the efficacy of 6 primary endpoints and other relevant indicators while also documenting the incidence of AEs. The aim was to provide a comprehensive assessment of the efficacy and safety of ADA therapy for PsA. Furthermore, all studies included in this meta-analysis were phase II and phase III RCTs. However, this study has several limitations. First, the number of included studies was limited, resulting in a relatively small sample size. Second, the treatment protocols varied across the studies, leading to considerable heterogeneity in the overall approach and potentially influencing our findings. Although a random-effects model was employed to some extent to improve the reliability of the conclusions, further validation through additional clinical trials is required. A key limitation of this meta-analysis is the potential inflation of type I error due to multiple comparisons arising from the evaluation of several efficacy endpoints (ACR20/50/70, PASI75/90/100) and multiple subgroup analyses. These analyses should therefore be interpreted as exploratory rather than confirmatory. Although this may increase the risk of false-positive findings, the consistency of treatment effects across primary outcomes and the robustness demonstrated in sensitivity analyses support the overall reliability of the conclusions. A key limitation of this meta-analysis is the substantial clinical heterogeneity across the included studies. The patient populations varied in terms of prior treatment exposure (biologic-naïve vs biologic-experienced), disease severity, and concomitant use of conventional DMARDs. These differences may have contributed to between-study variability and could potentially influence the magnitude of treatment effects. Although subgroup analyses were performed to explore some of these factors, residual confounding cannot be excluded. Therefore, the pooled estimates should be interpreted with caution in the context of heterogeneous study populations.

## 5. Conclusion

ADA demonstrated beneficial efficacy in treating PsA, as evidenced by improvements in ACR20, ACR50, ACR70, PASI75, PASI90, PASI100, and other relevant indicators. Although overall AEs were not increased, a significantly elevated risk of ALT and AST abnormalities was observed, indicating a potential signal of hepatotoxicity. Therefore, liver function monitoring should be considered during clinical use. A more detailed analysis of specific AEs highlighted potential concerns regarding hepatotoxicity. This suggested that ADA could potentially serve as a promising treatment option for patients with PsA.

## Author contributions

**Conceptualization:** Zhiyue Cai, Yue Zhang.

**Data curation:** Zhiyue Cai, Le Yan.

**Formal analysis:** Le Yan, Yue Zhang.

**Funding acquisition:** Zhiyue Cai, Yue Zhang.

**Investigation:** Le Yan.

**Methodology:** Yue Zhang.

**Project administration:** Le Yan.

**Resources:** Zhiyue Cai, Le Yan, Yue Zhang.

**Software:** Zhiyue Cai, Le Yan, Yue Zhang.

**Validation:** Zhiyue Cai, Le Yan, Yue Zhang.

**Visualization:** Zhiyue Cai, Le Yan, Yue Zhang.

**Writing – original draft:** Zhiyue Cai, Le Yan, Yue Zhang.























































## References

[1] Scher JU Ogdie A Merola JF Ritchlin C. Preventing psoriatic arthritis: focusing on patients with psoriasis at increased risk of transition. Nat Rev Rheumatol. 2019;15:153–66. 30742092 10.1038/s41584-019-0175-0

[2] López-Ferrer A Laiz A Puig L. Psoriatic arthritis. Med Clin (Barc). 2022;159:40–6. 35525675 10.1016/j.medcli.2022.01.024

[3] Popadic S Ramic Z Medenica L Pravica V Popadic D. IL-23R gene polymorphism rs2201841 is associated with psoriatic arthritis. Int J Immunogenet. 2014;41:335–7. 24910145 10.1111/iji.12127

[4] Duffin KC Freeny IC Schrodi SJ. Association between IL13 polymorphisms and psoriatic arthritis is modified by smoking. J Invest Dermatol. 2009;129:2777–83. 19554022 10.1038/jid.2009.169

[5] Azuaga AB Ramírez J Cañete JD. Psoriatic arthritis: pathogenesis and targeted therapies. Int J Mol Sci. 2023;24:4901. 36902329 10.3390/ijms24054901 PMC10003101

[6] Lee A Qiao Y Grigoriev G. Tumor necrosis factor α induces sustained signaling and a prolonged and unremitting inflammatory response in rheumatoid arthritis synovial fibroblasts. Arthritis Rheum. 2013;65:928–38. 23335080 10.1002/art.37853 PMC3618592

[7] Liu M Wu C Wu C. Immune cells differentiation in osteoarthritic cartilage damage: friends or foes? Front Immunol. 2025;16:1545284. 40201177 10.3389/fimmu.2025.1545284 PMC11975574

[8] Kitaura H Marahleh A Ohori F. Role of the interaction of tumor necrosis factor-α and tumor necrosis factor receptors 1 and 2 in bone-related cells. Int J Mol Sci. 2022;23:1481. 35163403 10.3390/ijms23031481 PMC8835906

[9] Phan QT Liu R Tan WH. Macrophages switch to an osteo-modulatory profile upon RANKL induction in a medaka (Oryzias latipes) osteoporosis model. JBMR Plus. 2020;4:e10409. 33210062 10.1002/jbm4.10409 PMC7657398

[10] Kerschbaumer A Fenzl KH Erlacher L Aletaha D. An overview of psoriatic arthritis - epidemiology, clinical features, pathophysiology and novel treatment targets. Wien Klin Wochenschr. 2016;128:791–5. 27822746 10.1007/s00508-016-1111-9 PMC5104808

[11] Gossec L Kerschbaumer A Ferreira RJO. EULAR recommendations for the management of psoriatic arthritis with pharmacological therapies: 2023 update. Ann Rheum Dis. 2024;83:706–19. 38499325 10.1136/ard-2024-225531 PMC11103320

[12] Ogdie A Coates LC Gladman DD. Treatment guidelines in psoriatic arthritis. Rheumatology (Oxford). 2020;59:i37–46. 32159790 10.1093/rheumatology/kez383 PMC7065461

[13] Ianculescu I Weisman MH. The role of methotrexate in psoriatic arthritis: what is the evidence? Clin Exp Rheumatol. 2015;33:S94–7. 26470718

[14] Sundanum S Orr C Veale D. Targeted therapies in psoriatic arthritis-an update. Int J Mol Sci. 2023;24:6384. 37047357 10.3390/ijms24076384 PMC10094037

[15] Mohd Noor AA Azlan M Mohd Redzwan N. Orchestrated cytokines mediated by biologics in psoriasis and its mechanisms of action. Biomedicines. 2022;10:498. 35203707 10.3390/biomedicines10020498 PMC8962336

[16] Lubrano E Scriffignano S Perrotta FM. Sequencing of biologic and target synthetic disease-modifying anti-rheumatic drugs in psoriatic arthritis: are we ready to redefine the treatment strategy? A perspective. Rheumatol Ther. 2023;10:301–6. 36495403 10.1007/s40744-022-00514-0 PMC10011266

[17] Watson M Tillett W Jadon D. The protocol of a clinical effectiveness trial comparing standard step-up care, early combination DMARD therapy and early use of TNF inhibitors for the treatment of moderate to severe psoriatic arthritis: the 3-arm parallel group SPEED randomized controlled trial. Ther Adv Musculoskelet Dis. 2024;16:1759720X241240913. 10.1177/1759720X241240913 PMC11143811 38826570

[18] Leone GM Mangano K Petralia MC Nicoletti F Fagone P. Past, present and (foreseeable) future of biological anti-TNF alpha therapy. J Clin Med. 2023;12:1630. 36836166 10.3390/jcm12041630 PMC9963154

[19] Reimold AM. The role of adalimumab in rheumatic and autoimmune disorders: comparison with other biologic agents. Open Access Rheumatol. 2012;4:33–47. 27790010 10.2147/OARRR.S14569 PMC5045097

[20] Hu S Liang S Guo H. Comparison of the inhibition mechanisms of adalimumab and infliximab in treating tumor necrosis factor α-associated diseases from a molecular view. J Biol Chem. 2013;288:27059–67. 23943614 10.1074/jbc.M113.491530 PMC3779706

[21] Tamai H Ikeda K Miyamoto T. Reduced versus maximum tolerated methotrexate dose concomitant with adalimumab in patients with rheumatoid arthritis (MIRACLE): a randomised, open-label, non-inferiority trial. Lancet Rheumatol. 2023;5:e215–24. 38251524 10.1016/S2665-9913(23)00070-X

[22] van der Heijde D Ramiro S Landewé R. 2016 update of the ASAS-EULAR management recommendations for axial spondyloarthritis. Ann Rheum Dis. 2017;76:978–91. 28087505 10.1136/annrheumdis-2016-210770

[23] Warren RB Blauvelt A Bagel J. Bimekizumab versus adalimumab in plaque psoriasis. N Engl J Med. 2021;385:130–41. 33891379 10.1056/NEJMoa2102388

[24] Panaccione R Colombel J-F Sandborn WJ. Adalimumab maintains remission of Crohn’s disease after up to 4 years of treatment: data from CHARM and ADHERE. Aliment Pharmacol Ther. 2013;38:1236–47. 24134498 10.1111/apt.12499 PMC4670480

[25] Dai C Huang YH Jiang M. Successful treatment of ulcerative colitis, rectovaginal fistula, and skin erythema nodule with adalimumab. Inflamm Bowel Dis. 2022;28:e36–7. 34718590 10.1093/ibd/izab260

[26] Pichi F Smith SD AlAli SH Neri P. Adalimumab drug monitoring and treatment adjustment to drug antibodies in noninfectious uveitis. Am J Ophthalmol. 2024;268:306–11. 39271091 10.1016/j.ajo.2024.09.008

[27] Angeles-Han ST Ringold S Beukelman T. 2019 American College of Rheumatology/Arthritis Foundation guideline for the screening, monitoring, and treatment of juvenile idiopathic arthritis-associated uveitis. Arthritis Care Res (Hoboken). 2019;71:703–16. 31021540 10.1002/acr.23871 PMC6777949

[28] Sator P. Safety and tolerability of adalimumab for the treatment of psoriasis: a review summarizing 15 years of real-life experience. Ther Adv Chronic Dis. 2018;9:147–58. 30065812 10.1177/2040622318772705 PMC6052502

[29] Montezuma T Probst LF Almeida MO. Effectiveness and safety of biological and target synthetic drugs treatment for psoriatic arthritis: a systematic review with network meta-analysis. Adv Rheumatol. 2024;64:21. 38515177 10.1186/s42358-024-00361-3 PMC13488810

[30] Moher D Shamseer L Clarke M. Preferred Reporting Items for Systematic Review and Meta-Analysis Protocols (PRISMA-P) 2015 statement. Syst Rev. 2015;4:1. 25554246 10.1186/2046-4053-4-1 PMC4320440

[31] Sterne JAC Savović J Page MJ. RoB 2: a revised tool for assessing risk of bias in randomised trials. BMJ. 2019;366:l4898. 31462531 10.1136/bmj.l4898

[32] Mease PJ Gladman DD Ritchlin CT. Adalimumab for the treatment of patients with moderately to severely active psoriatic arthritis: results of a double-blind, randomized, placebo-controlled trial. Arthritis Rheum. 2005;52:3279–89. 16200601 10.1002/art.21306

[33] Gordon KB Langley RG Leonardi C. Clinical response to adalimumab treatment in patients with moderate to severe psoriasis: double-blind, randomized controlled trial and open-label extension study. J Am Acad Dermatol. 2006;55:598–606. 17010738 10.1016/j.jaad.2006.05.027

[34] Genovese MC Mease PJ Thomson GTD. Safety and efficacy of adalimumab in treatment of patients with psoriatic arthritis who had failed disease modifying antirheumatic drug therapy. J Rheumatol. 2007;34:1040–50. 17444593

[35] Menter A Tyring SK Gordon K. Adalimumab therapy for moderate to severe psoriasis: a randomized, controlled phase III trial. J Am Acad Dermatol. 2008;58:106–15. 17936411 10.1016/j.jaad.2007.09.010

[36] Revicki D Willian MK Saurat J-H. Impact of adalimumab treatment on health-related quality of life and other patient-reported outcomes: results from a 16-week randomized controlled trial in patients with moderate to severe plaque psoriasis. Br J Dermatol. 2008;158:549–57. 18047521 10.1111/j.1365-2133.2007.08236.x

[37] van Kuijk AW Gerlag DM Vos K. A prospective, randomised, placebo-controlled study to identify biomarkers associated with active treatment in psoriatic arthritis: effects of adalimumab treatment on synovial tissue. Ann Rheum Dis. 2009;68:1303–9. 18647851 10.1136/ard.2008.091389 PMC2703703

[38] Paramarta JE De Rycke L Heijda TF. Efficacy and safety of adalimumab for the treatment of peripheral arthritis in spondyloarthritis patients without ankylosing spondylitis or psoriatic arthritis. Ann Rheum Dis. 2013;72:1793–9. 23139265 10.1136/annrheumdis-2012-202245

[39] Mease P Sieper J Van den Bosch F Rahman P Karunaratne PM Pangan AL. Randomized controlled trial of adalimumab in patients with nonpsoriatic peripheral spondyloarthritis. Arthritis Rheumatol. 2015;67:914–23. 25545240 10.1002/art.39008 PMC4409087

[40] Mease PJ van der Heijde D Ritchlin CT. Ixekizumab, an interleukin-17A specific monoclonal antibody, for the treatment of biologic-naive patients with active psoriatic arthritis: results from the 24-week randomised, double-blind, placebo-controlled and active (adalimumab)-controlled period of the phase III trial SPIRIT-P1. Ann Rheum Dis. 2017;76:79–87. 27553214 10.1136/annrheumdis-2016-209709 PMC5264219

[41] Mease P Hall S FitzGerald O. Tofacitinib or adalimumab versus placebo for psoriatic arthritis. N Engl J Med. 2017;377:1537–50. 29045212 10.1056/NEJMoa1615975

[42] Mease PJ Genovese MC Weinblatt ME. Phase II study of ABT-122, a tumor necrosis factor- and interleukin-17A-targeted dual variable domain immunoglobulin, in patients with psoriatic arthritis with an inadequate response to methotrexate. Arthritis Rheumatol. 2018;70:1778–89. 29855175 10.1002/art.40579 PMC6221045

[43] Gottlieb AB Strand V Kishimoto M. Ixekizumab improves patient-reported outcomes up to 52 weeks in bDMARD-naïve patients with active psoriatic arthritis (SPIRIT-P1). Rheumatology (Oxford). 2018;57:1777–88. 29945203 10.1093/rheumatology/key161 PMC6152421

[44] Strand V de Vlam K Covarrubias-Cobos JA. Tofacitinib or adalimumab versus placebo: patient-reported outcomes from OPAL Broaden-a phase III study of active psoriatic arthritis in patients with an inadequate response to conventional synthetic disease-modifying antirheumatic drugs. RMD Open. 2019;5:e000806. 30713721 10.1136/rmdopen-2018-000806 PMC6340575

[45] McInnes IB Anderson JK Magrey M. Trial of upadacitinib and adalimumab for psoriatic arthritis. N Engl J Med. 2021;384:1227–39. 33789011 10.1056/NEJMoa2022516

[46] Strand V Mease PJ Soriano ER. Improvement in patient-reported outcomes in patients with psoriatic arthritis treated with upadacitinib versus placebo or adalimumab: results from SELECT-PsA 1. Rheumatol Ther. 2021;8:1789–808. 34636026 10.1007/s40744-021-00379-9 PMC8572257

[47] McInnes IB Asahina A Coates LC. Bimekizumab in patients with psoriatic arthritis, naive to biologic treatment: a randomised, double-blind, placebo-controlled, phase 3 trial (BE OPTIMAL). Lancet. 2023;401:25–37. 36493791 10.1016/S0140-6736(22)02302-9

[48] Ritchlin CT Coates LC McInnes IB. Bimekizumab treatment in biologic DMARD-naïve patients with active psoriatic arthritis: 52-week efficacy and safety results from the phase III, randomised, placebo-controlled, active reference BE OPTIMAL study. Ann Rheum Dis. 2023;82:1404–14. 37696588 10.1136/ard-2023-224431 PMC10579478

[49] Singh JA Guyatt G Ogdie A. Special article: 2018 American College of Rheumatology/National Psoriasis Foundation guideline for the treatment of psoriatic arthritis. Arthritis Rheumatol. 2019;71:5–32. 30499246 10.1002/art.40726 PMC8218333

[50] Ogdie A Coates LC Mease P. Measuring outcomes in psoriatic arthritis. Arthritis Care Res (Hoboken). 2020;72:82–109. 33091263 10.1002/acr.24242 PMC8528225

[51] Motyl G Krupka WM Maślińska M. The problem of residual pain in the assessment of rheumatoid arthritis activity. Reumatologia. 2024;62:176–86. 39055728 10.5114/reum/189779 PMC11267660

[52] Kay J Jaworski J Wojciechowski R. Efficacy and safety of biosimilar CT-P17 versus reference adalimumab in subjects with rheumatoid arthritis: 24-week results from a randomized study. Arthritis Res Ther. 2021;23:51. 33546755 10.1186/s13075-020-02394-7 PMC7863328

[53] Ward MM Guthrie LC Alba MI. Clinically important changes in individual and composite measures of rheumatoid arthritis activity: thresholds applicable in clinical trials. Ann Rheum Dis. 2015;74:1691–6. 24794149 10.1136/annrheumdis-2013-205079 PMC4216638

[54] Strand V Elaine Husni M Betts KA. Network meta-analysis and cost per responder of targeted immunomodulators in the treatment of active psoriatic arthritis. BMC Rheumatol. 2018;2:3. 30886954 10.1186/s41927-018-0011-1 PMC6390550

[55] Gordon KB Reich K Crowley JJ. Disease activity and treatment efficacy using patient-level Psoriasis Area and Severity Index scores from tildrakizumab phase 3 clinical trials. J Dermatolog Treat. 2022;33:219–28. 32349565 10.1080/09546634.2020.1747590

[56] Gerdes S Körber A Biermann M Karnthaler C Reinhardt M. Absolute and relative Psoriasis Area and Severity Index (PASI) treatment goals and their association with health-related quality of life. J Dermatolog Treat. 2020;31:470–5. 32202943 10.1080/09546634.2020.1746734

[57] Chandrasekharan UM Kaur R Harvey JE. TNFR2 depletion reduces psoriatic inflammation in mice by downregulating specific dendritic cell populations in lymph nodes and inhibiting IL-23/IL-17 pathways. J Invest Dermatol. 2022;142:2159–72.e9. 35090950 10.1016/j.jid.2021.12.036 PMC9314460

[58] Boutet MA Nerviani A Gallo Afflitto G Pitzalis C. Role of the IL-23/IL-17 axis in psoriasis and psoriatic arthritis: the clinical importance of its divergence in skin and joints. Int J Mol Sci. 2018;19:530. 29425183 10.3390/ijms19020530 PMC5855752

[59] Wang CQF Akalu YT Suarez-Farinas M. IL-17 and TNF synergistically modulate cytokine expression while suppressing melanogenesis: potential relevance to psoriasis. J Invest Dermatol. 2013;133:2741–52. 23732752 10.1038/jid.2013.237 PMC3830693

[60] Mease PJ. Measures of psoriatic arthritis: tender and swollen joint assessment, Psoriasis Area and Severity Index (PASI), Nail Psoriasis Severity Index (NAPSI), Modified Nail Psoriasis Severity Index (mNAPSI), Mander/Newcastle Enthesitis Index (MEI), Leeds Enthesitis Index (LEI), Spondyloarthritis Research Consortium of Canada (SPARCC), Maastricht Ankylosing Spondylitis Enthesis Score (MASES), Leeds Dactylitis Index (LDI), Patient Global for Psoriatic Arthritis, Dermatology Life Quality Index (DLQI), Psoriatic Arthritis Quality of Life (PsAQOL), Functional Assessment of Chronic Illness Therapy-Fatigue (FACIT-F), Psoriatic Arthritis Response Criteria (PsARC), Psoriatic Arthritis Joint Activity Index (PsAJAI), Disease Activity in Psoriatic Arthritis (DAPSA), and Composite Psoriatic Disease Activity Index (CPDAI). Arthritis Care Res (Hoboken). 2011;63:S64–85. 22588772 10.1002/acr.20577

[61] Orbai AM Weitz J Siegel EL. Systematic review of treatment effectiveness and outcome measures for enthesitis in psoriatic arthritis. J Rheumatol. 2014;41:2290–4. 25362713 10.3899/jrheum.140878

[62] Coates LC Strand V Wilson H. Measurement properties of the minimal disease activity criteria for psoriatic arthritis. RMD Open. 2019;5:e001002. 31565243 10.1136/rmdopen-2019-001002 PMC6744081

[63] Mease PJ Heckaman M Kary S Kupper H. Application and modifications of minimal disease activity measures for patients with psoriatic arthritis treated with adalimumab: subanalyses of ADEPT. J Rheumatol. 2013;40:647–52. 23504383 10.3899/jrheum.120970

