# Supplementary material for: Efficacy and safety evaluation of adalimumab for psoriatic arthritis: A meta-analysis
Source: Medicine (Baltimore). 2026 Aug 21;105(34):e50295. doi: 10.1097/MD.0000000000050295 (PMC13502872; doi:10.1097/MD.0000000000050295)
Supplement: Supplementary file 1 [file medi-105-e50295-s001.pdf]

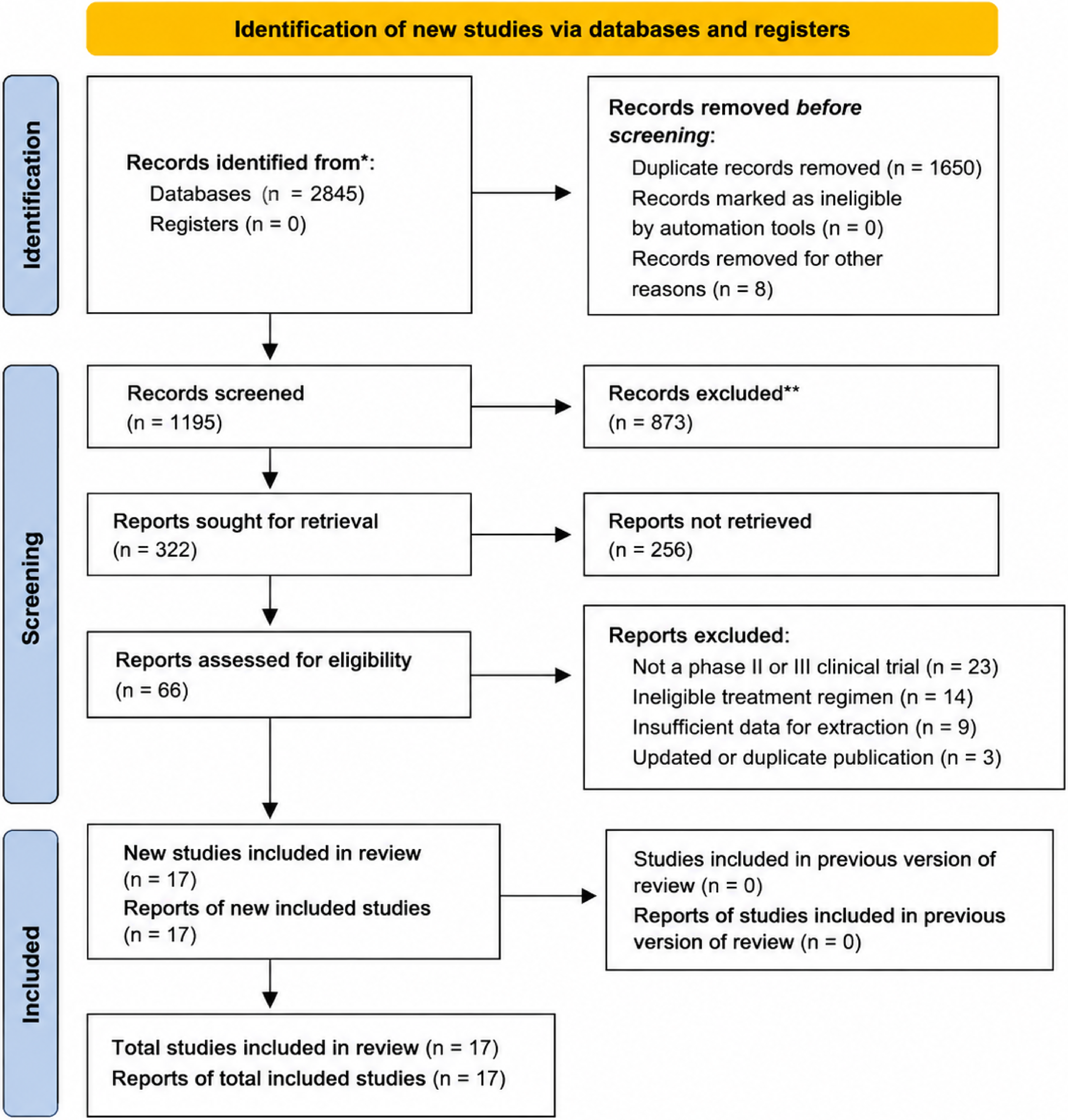
Identification of new studies via databases and registers
Identification
Records identified from*:
Databases (n = 2845)
Registers (n = 0)
Records removed before screening:
Duplicate records removed (n = 1650)
Records marked as ineligible by automation tools (n = 0)
Records removed for other reasons (n = 8)
Screening
Records screened (n = 1195)
Records excluded** (n = 873)
Reports sought for retrieval (n = 322)
Reports not retrieved (n = 256)
Reports assessed for eligibility (n = 66)
Reports excluded:
Not a phase II or III clinical trial (n = 23)
Ineligible treatment regimen (n = 14)
Insufficient data for extraction (n = 9)
Updated or duplicate publication (n = 3)
Included
New studies included in review (n = 17)
Reports of new included studies (n = 17)
Studies included in previous version of review (n = 0)
Reports of studies included in previous version of review (n = 0)
Total studies included in review (n = 17)
Reports of total included studies (n = 17)
