# Supplementary material for: Efficacy and safety evaluation of adalimumab for psoriatic arthritis: A meta-analysis
Source: Medicine (Baltimore). 2026 Aug 21;105(34):e50295. doi: 10.1097/MD.0000000000050295 (PMC13502872; doi:10.1097/MD.0000000000050295)
Supplement: Supplementary file 3 [file medi-105-e50295-s004.pdf]

	Random sequence generation (selection bias)	Allocation concealment (selection bias)	Blinding of participants and personnel (performance bias)	Blinding of outcome assessment (detection bias)	Incomplete outcome data (attrition bias)	Selective reporting (reporting bias)	Other bias
Genovese 2007	+	?	+	?	+	+	?
Gordon 2006	?	?	+	?	+	+	+
Gottlieb 2018	?	?	+	?	+	+	+
McInnes 2021	+	?	+	?	+	+	+
McInnes 2023	+	?	+	?	+	+	+
Mease 2015	+	?	+	?	+	+	+
Mease 2017	+	+	+	?	+	+	+
Mease 2017-a	+	+	+	?	+	+	+
Mease 2018	?	+	+	?	+	+	+
Menter 2008	?	+	+	?	+	+	+
Paramarta 2013	?	?	+	?	+	+	+
Revicki 2008	?	?	+	?	+	+	+
Ritchlin 2023	?	?	+	?	+	+	+
Strand 2019	?	?	+	?	+	+	+
Strand 2021	?	?	+	?	+	+	+
van Kuijk AW 2009	?	?	+	+	+	+	+
