# Supplementary material for: Efficacy and safety evaluation of adalimumab for psoriatic arthritis: A meta-analysis
Source: Medicine (Baltimore). 2026 Aug 21;105(34):e50295. doi: 10.1097/MD.0000000000050295 (PMC13502872; doi:10.1097/MD.0000000000050295)
Supplement: Supplementary file 5 [file medi-105-e50295-s006.pdf]

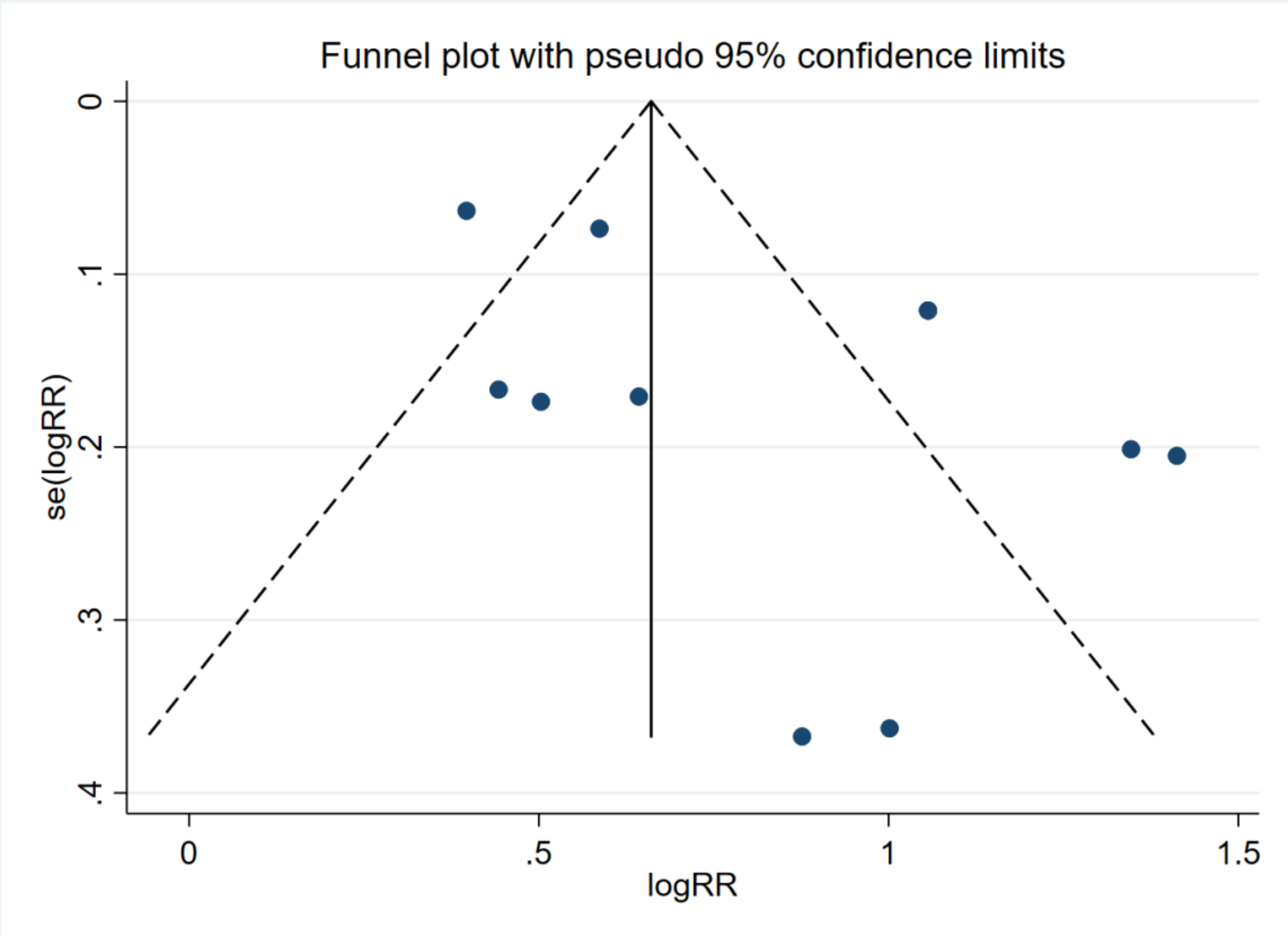

Funnel plot with pseudo 95% confidence limits
0
.1
.2
.3
.4
se(logRR)
0
.5
1
1.5
logRR
