# Supplementary material for: Efficacy and safety evaluation of adalimumab for psoriatic arthritis: A meta-analysis
Source: Medicine (Baltimore). 2026 Aug 21;105(34):e50295. doi: 10.1097/MD.0000000000050295 (PMC13502872; doi:10.1097/MD.0000000000050295)
Supplement: Supplementary file 6 [file medi-105-e50295-s007.pdf]

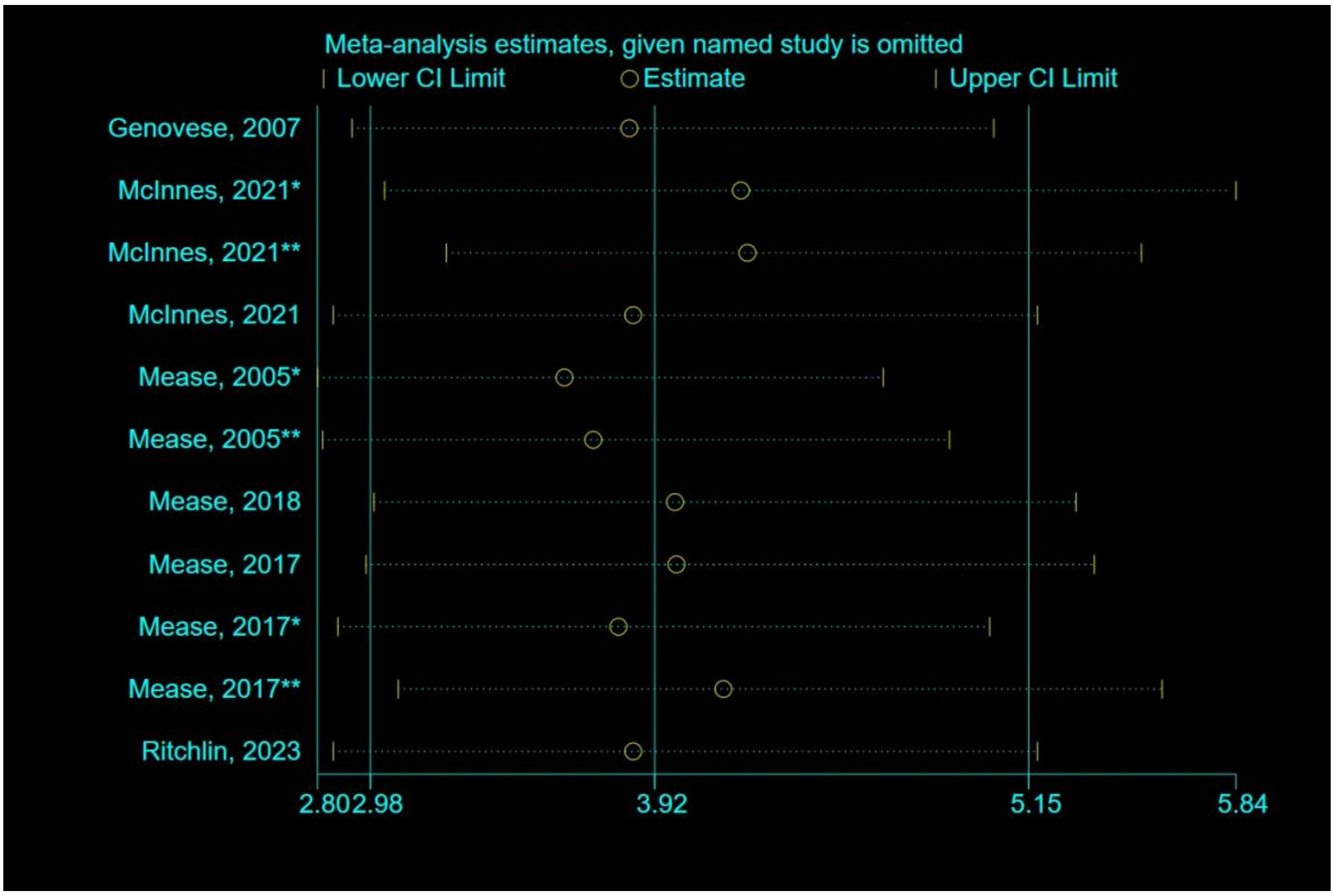

Meta-analysis estimates, given named study is omitted
Lower CI Limit
Estimate
Upper CI Limit
Genovese, 2007
McInnes, 2021*
McInnes, 2021**
McInnes, 2021
Mease, 2005*
Mease, 2005**
Mease, 2018
Mease, 2017
Mease, 2017*
Mease, 2017**
Ritchlin, 2023
2.80
2.98
3.92
5.15
5.84
