# Supplementary material for: Efficacy and safety evaluation of adalimumab for psoriatic arthritis: A meta-analysis
Source: Medicine (Baltimore). 2026 Aug 21;105(34):e50295. doi: 10.1097/MD.0000000000050295 (PMC13502872; doi:10.1097/MD.0000000000050295)
Supplement: Supplementary file 9 [file medi-105-e50295-s010.pdf]

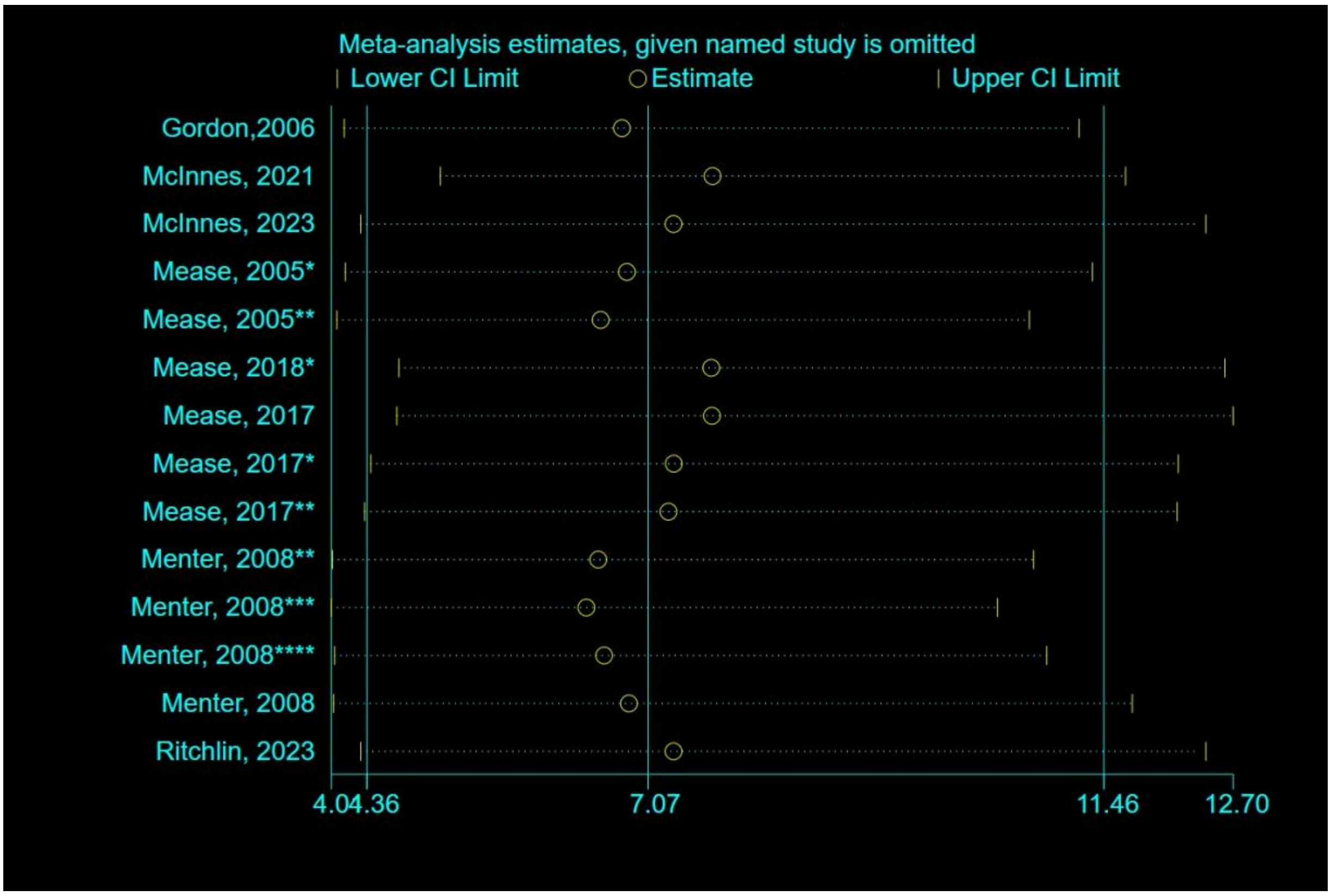
Meta-analysis estimates, given named study is omitted
Lower CI Limit
Estimate
Upper CI Limit
Gordon,2006
McInnes, 2021
McInnes, 2023
Mease, 2005*
Mease, 2005**
Mease, 2018*
Mease, 2017
Mease, 2017*
Mease, 2017**
Menter, 2008**
Menter, 2008***
Menter, 2008****
Menter, 2008
Ritchlin, 2023
4.0
4.36
7.07
11.46
12.70
