# Supplementary material for: Efficacy and safety evaluation of adalimumab for psoriatic arthritis: A meta-analysis
Source: Medicine (Baltimore). 2026 Aug 21;105(34):e50295. doi: 10.1097/MD.0000000000050295 (PMC13502872; doi:10.1097/MD.0000000000050295)
Supplement: Supplementary file 11 [file medi-105-e50295-s012.pdf]

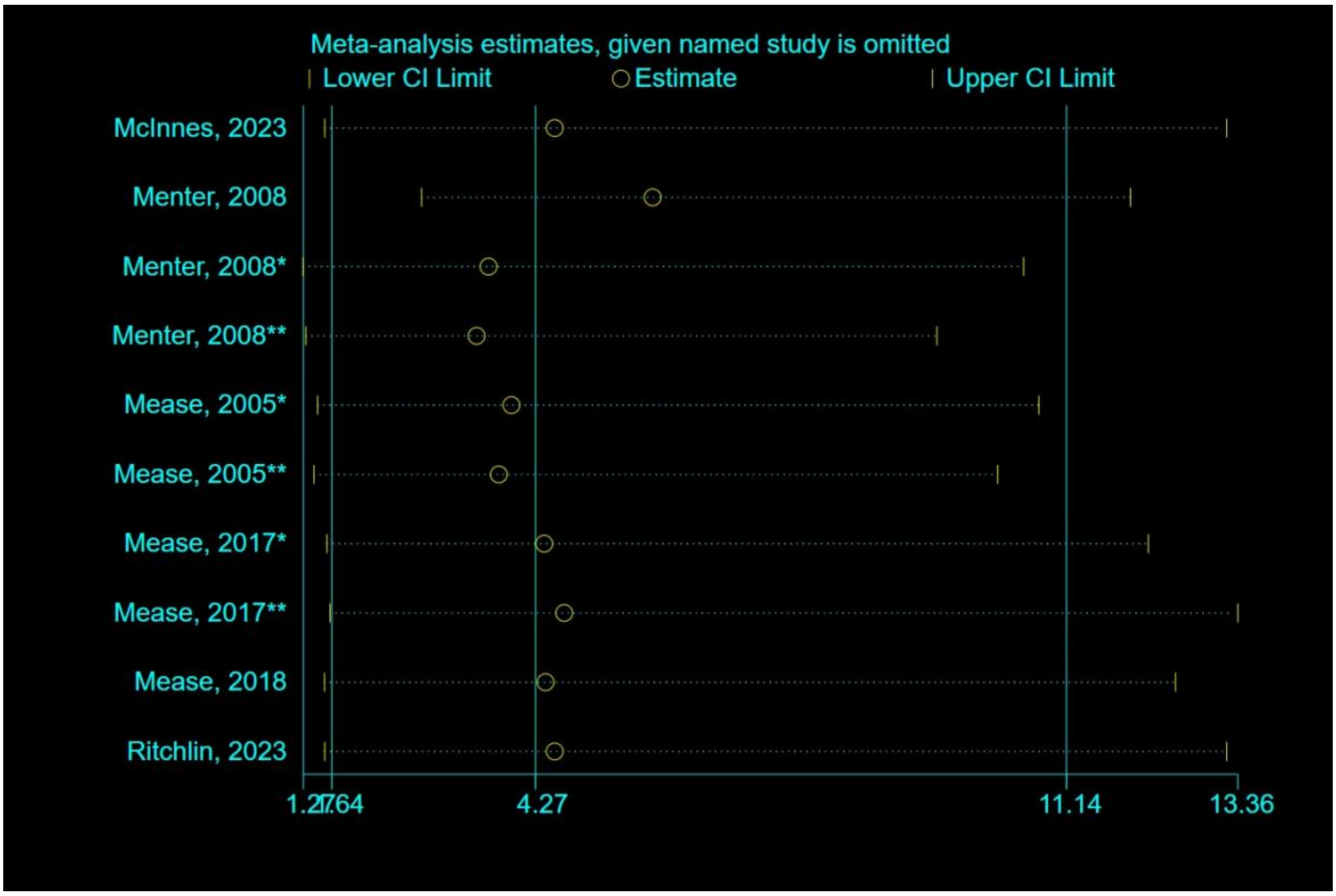

Meta-analysis estimates, given named study is omitted
Lower CI Limit
Estimate
Upper CI Limit
McInnes, 2023
Menter, 2008
Menter, 2008*
Menter, 2008**
Mease, 2005*
Mease, 2005**
Mease, 2017*
Mease, 2017**
Mease, 2018
Ritchlin, 2023
1.27
1.64
4.27
11.14
13.36
