# Supplementary material for: Efficacy and safety evaluation of adalimumab for psoriatic arthritis: A meta-analysis
Source: Medicine (Baltimore). 2026 Aug 21;105(34):e50295. doi: 10.1097/MD.0000000000050295 (PMC13502872; doi:10.1097/MD.0000000000050295)
Supplement: Supplementary file 13 [file medi-105-e50295-s014.pdf]

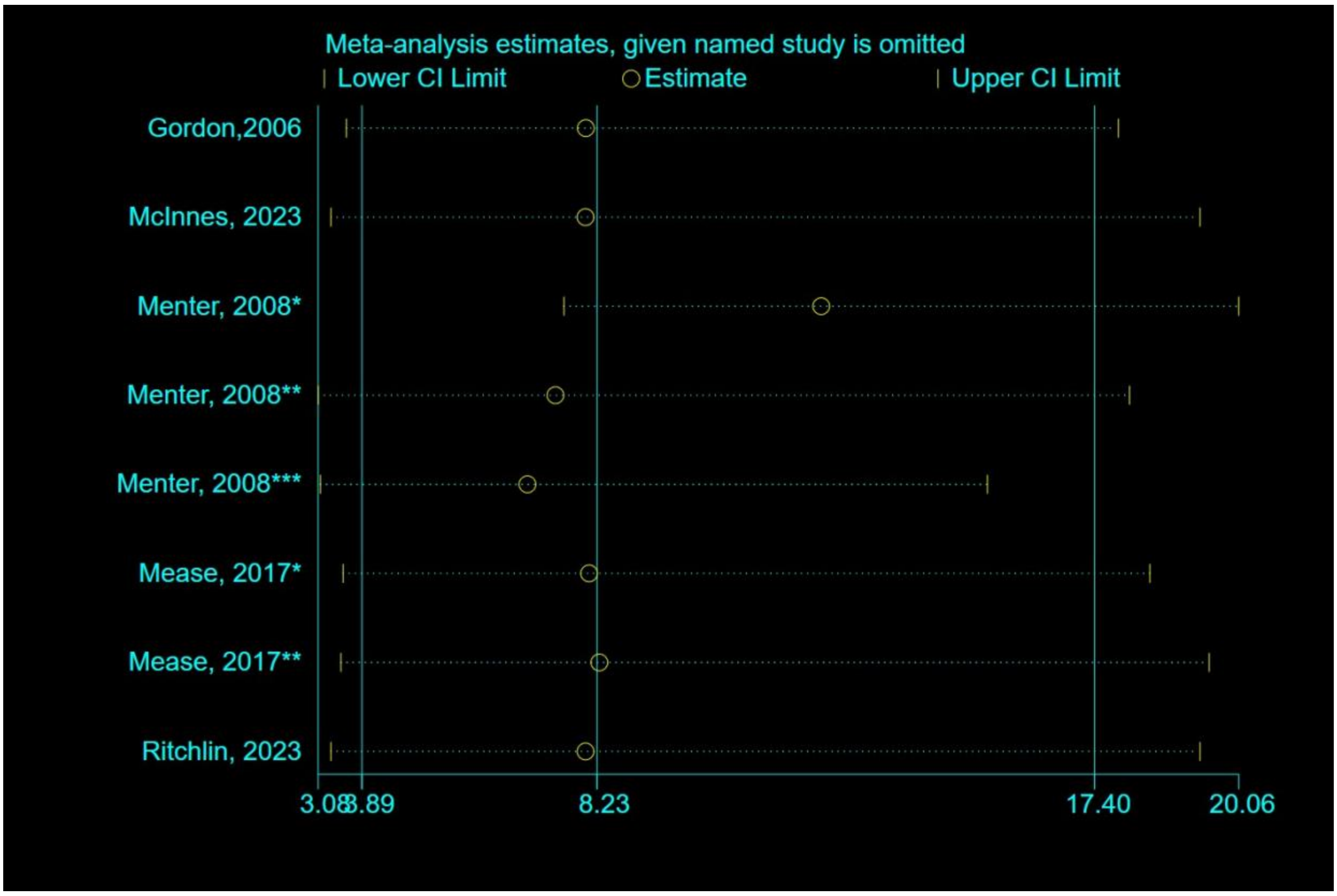
Meta-analysis estimates, given named study is omitted
Lower CI Limit
Estimate
Upper CI Limit
Gordon,2006
McInnes, 2023
Menter, 2008*
Menter, 2008**
Menter, 2008***
Mease, 2017*
Mease, 2017**
Ritchlin, 2023
3.08
3.89
8.23
17.40
20.06
