# Supplementary material for: Efficacy and safety evaluation of adalimumab for psoriatic arthritis: A meta-analysis
Source: Medicine (Baltimore). 2026 Aug 21;105(34):e50295. doi: 10.1097/MD.0000000000050295 (PMC13502872; doi:10.1097/MD.0000000000050295)
Supplement: Supplementary file 15 [file medi-105-e50295-s016.pdf]

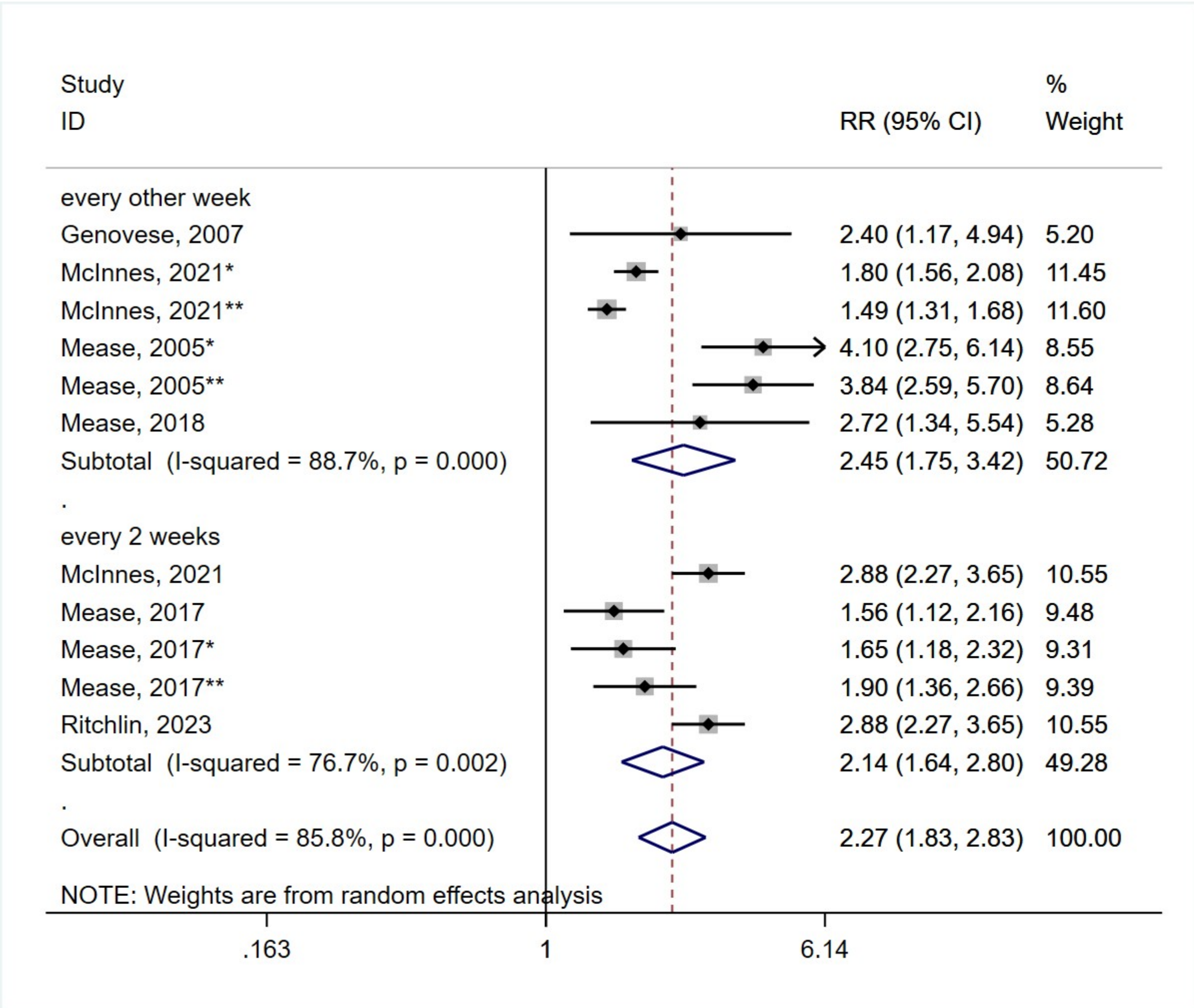
Study
ID
RR (95% CI)
%
Weight
every other week
Genovese, 2007
2.40 (1.17, 4.94)
5.20
McInnes, 2021*
1.80 (1.56, 2.08)
11.45
McInnes, 2021**
1.49 (1.31, 1.68)
11.60
Mease, 2005*
4.10 (2.75, 6.14)
8.55
Mease, 2005**
3.84 (2.59, 5.70)
8.64
Mease, 2018
2.72 (1.34, 5.54)
5.28
Subtotal (I-squared = 88.7%, p = 0.000)
2.45 (1.75, 3.42)
50.72
.
every 2 weeks
McInnes, 2021
2.88 (2.27, 3.65)
10.55
Mease, 2017
1.56 (1.12, 2.16)
9.48
Mease, 2017*
1.65 (1.18, 2.32)
9.31
Mease, 2017**
1.90 (1.36, 2.66)
9.39
Ritchlin, 2023
2.88 (2.27, 3.65)
10.55
Subtotal (I-squared = 76.7%, p = 0.002)
2.14 (1.64, 2.80)
49.28
.
Overall (I-squared = 85.8%, p = 0.000)
2.27 (1.83, 2.83)
100.00
NOTE: Weights are from random effects analysis
.163
1
6.14
