# Supplementary material for: Efficacy and safety evaluation of adalimumab for psoriatic arthritis: A meta-analysis
Source: Medicine (Baltimore). 2026 Aug 21;105(34):e50295. doi: 10.1097/MD.0000000000050295 (PMC13502872; doi:10.1097/MD.0000000000050295)
Supplement: Supplementary file 16 [file medi-105-e50295-s017.pdf]

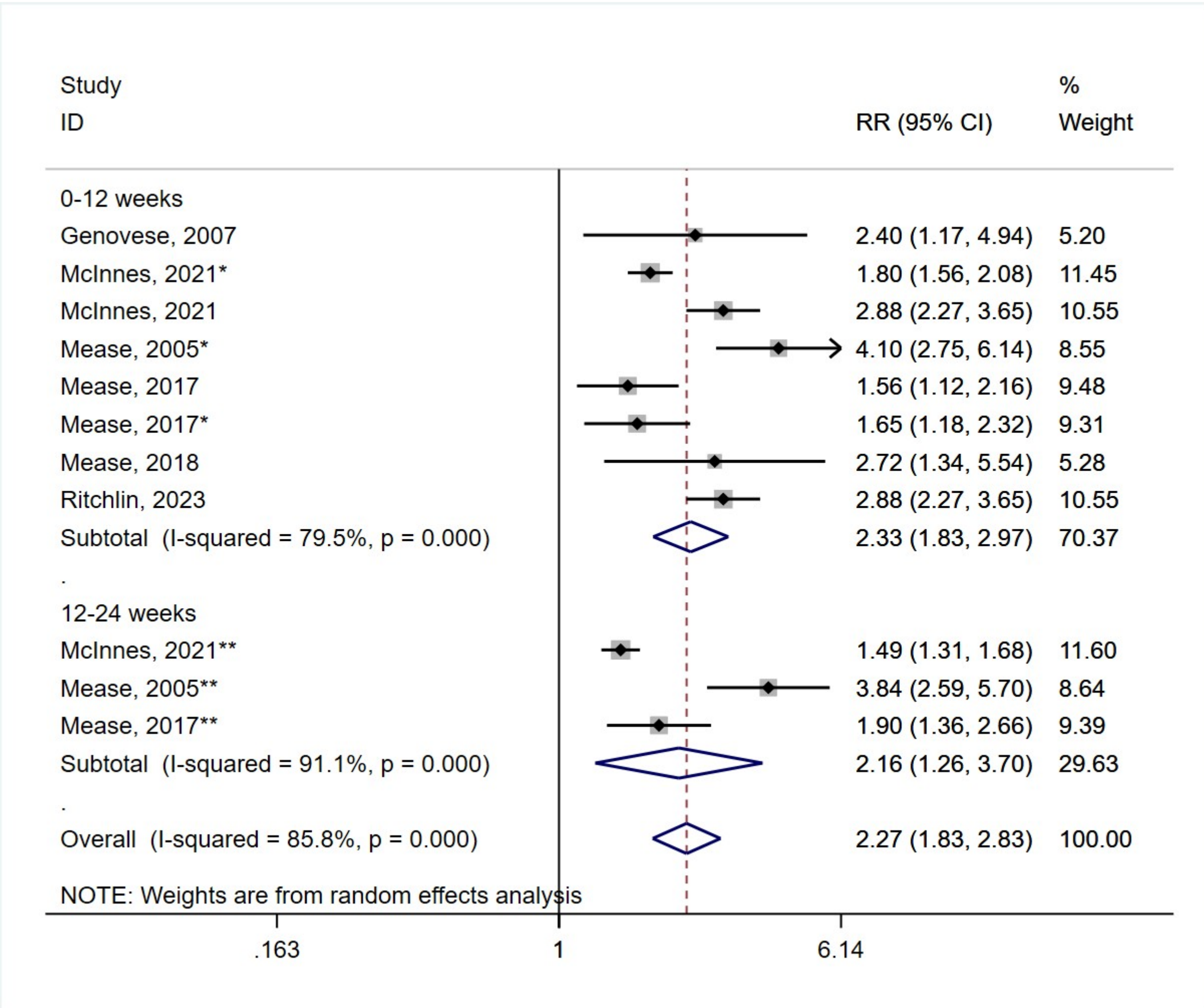
Study
ID
RR (95% CI)
%
Weight
0-12 weeks
Genovese, 2007 2.40 (1.17, 4.94) 5.20
McInnes, 2021* 1.80 (1.56, 2.08) 11.45
McInnes, 2021 2.88 (2.27, 3.65) 10.55
Mease, 2005* 4.10 (2.75, 6.14) 8.55
Mease, 2017 1.56 (1.12, 2.16) 9.48
Mease, 2017* 1.65 (1.18, 2.32) 9.31
Mease, 2018 2.72 (1.34, 5.54) 5.28
Ritchlin, 2023 2.88 (2.27, 3.65) 10.55
Subtotal (I-squared = 79.5%, p = 0.000) 2.33 (1.83, 2.97) 70.37
.
12-24 weeks
McInnes, 2021** 1.49 (1.31, 1.68) 11.60
Mease, 2005** 3.84 (2.59, 5.70) 8.64
Mease, 2017** 1.90 (1.36, 2.66) 9.39
Subtotal (I-squared = 91.1%, p = 0.000) 2.16 (1.26, 3.70) 29.63
.
Overall (I-squared = 85.8%, p = 0.000) 2.27 (1.83, 2.83) 100.00
NOTE: Weights are from random effects analysis
.163
1
6.14
