# Supplementary material for: Efficacy and safety evaluation of adalimumab for psoriatic arthritis: A meta-analysis
Source: Medicine (Baltimore). 2026 Aug 21;105(34):e50295. doi: 10.1097/MD.0000000000050295 (PMC13502872; doi:10.1097/MD.0000000000050295)
Supplement: Supplementary file 17 [file medi-105-e50295-s018.pdf]

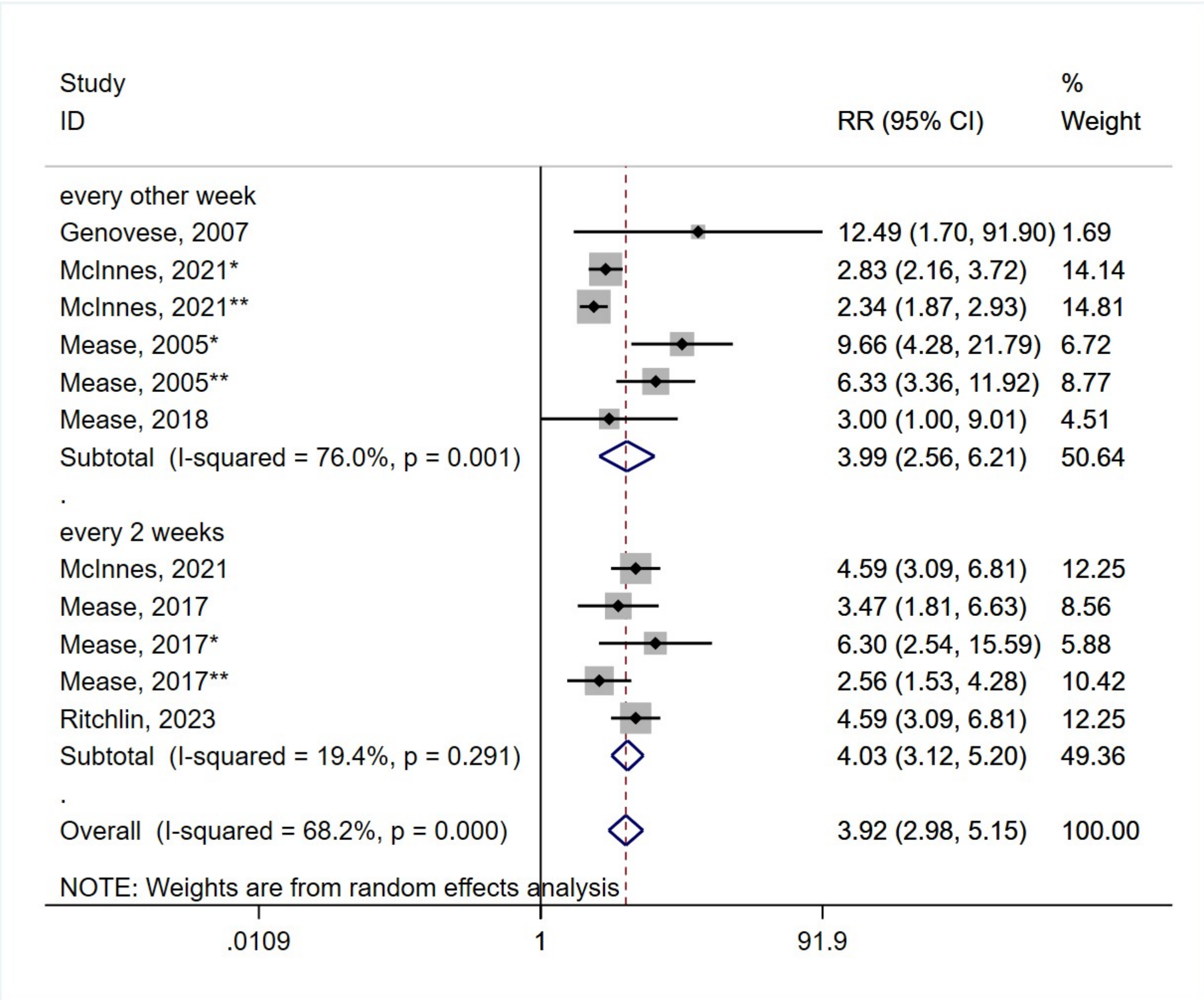
Study
ID
RR (95% CI)
%
Weight
every other week
Genovese, 2007
12.49 (1.70, 91.90) 1.69
McInnes, 2021*
2.83 (2.16, 3.72) 14.14
McInnes, 2021**
2.34 (1.87, 2.93) 14.81
Mease, 2005*
9.66 (4.28, 21.79) 6.72
Mease, 2005**
6.33 (3.36, 11.92) 8.77
Mease, 2018
3.00 (1.00, 9.01) 4.51
Subtotal (I-squared = 76.0%, p = 0.001)
3.99 (2.56, 6.21) 50.64
every 2 weeks
McInnes, 2021
4.59 (3.09, 6.81) 12.25
Mease, 2017
3.47 (1.81, 6.63) 8.56
Mease, 2017*
6.30 (2.54, 15.59) 5.88
Mease, 2017**
2.56 (1.53, 4.28) 10.42
Ritchlin, 2023
4.59 (3.09, 6.81) 12.25
Subtotal (I-squared = 19.4%, p = 0.291)
4.03 (3.12, 5.20) 49.36
Overall (I-squared = 68.2%, p = 0.000)
3.92 (2.98, 5.15) 100.00
NOTE: Weights are from random effects analysis
.0109
1
91.9
