# Supplementary material for: Efficacy and safety evaluation of adalimumab for psoriatic arthritis: A meta-analysis
Source: Medicine (Baltimore). 2026 Aug 21;105(34):e50295. doi: 10.1097/MD.0000000000050295 (PMC13502872; doi:10.1097/MD.0000000000050295)
Supplement: Supplementary file 18 [file medi-105-e50295-s019.pdf]

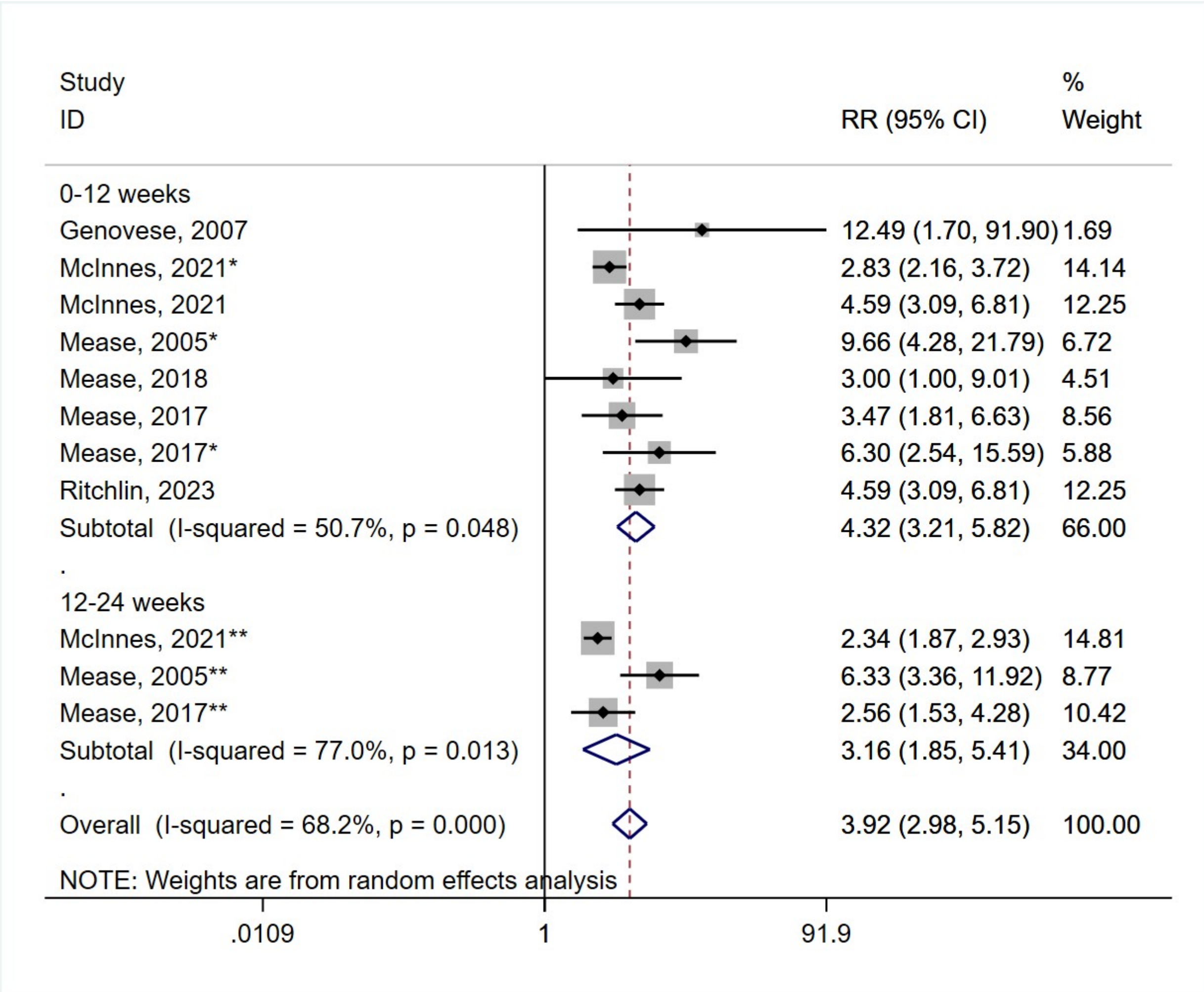
Study
ID
RR (95% CI)
%
Weight
0-12 weeks
Genovese, 2007
12.49 (1.70, 91.90)
1.69
McInnes, 2021*
2.83 (2.16, 3.72)
14.14
McInnes, 2021
4.59 (3.09, 6.81)
12.25
Mease, 2005*
9.66 (4.28, 21.79)
6.72
Mease, 2018
3.00 (1.00, 9.01)
4.51
Mease, 2017
3.47 (1.81, 6.63)
8.56
Mease, 2017*
6.30 (2.54, 15.59)
5.88
Ritchlin, 2023
4.59 (3.09, 6.81)
12.25
Subtotal (I-squared = 50.7%, p = 0.048)
4.32 (3.21, 5.82)
66.00
12-24 weeks
McInnes, 2021**
2.34 (1.87, 2.93)
14.81
Mease, 2005**
6.33 (3.36, 11.92)
8.77
Mease, 2017**
2.56 (1.53, 4.28)
10.42
Subtotal (I-squared = 77.0%, p = 0.013)
3.16 (1.85, 5.41)
34.00
Overall (I-squared = 68.2%, p = 0.000)
3.92 (2.98, 5.15)
100.00
NOTE: Weights are from random effects analysis
.0109
1
91.9
