# Supplementary material for: Efficacy and safety evaluation of adalimumab for psoriatic arthritis: A meta-analysis
Source: Medicine (Baltimore). 2026 Aug 21;105(34):e50295. doi: 10.1097/MD.0000000000050295 (PMC13502872; doi:10.1097/MD.0000000000050295)
Supplement: Supplementary file 19 [file medi-105-e50295-s020.pdf]

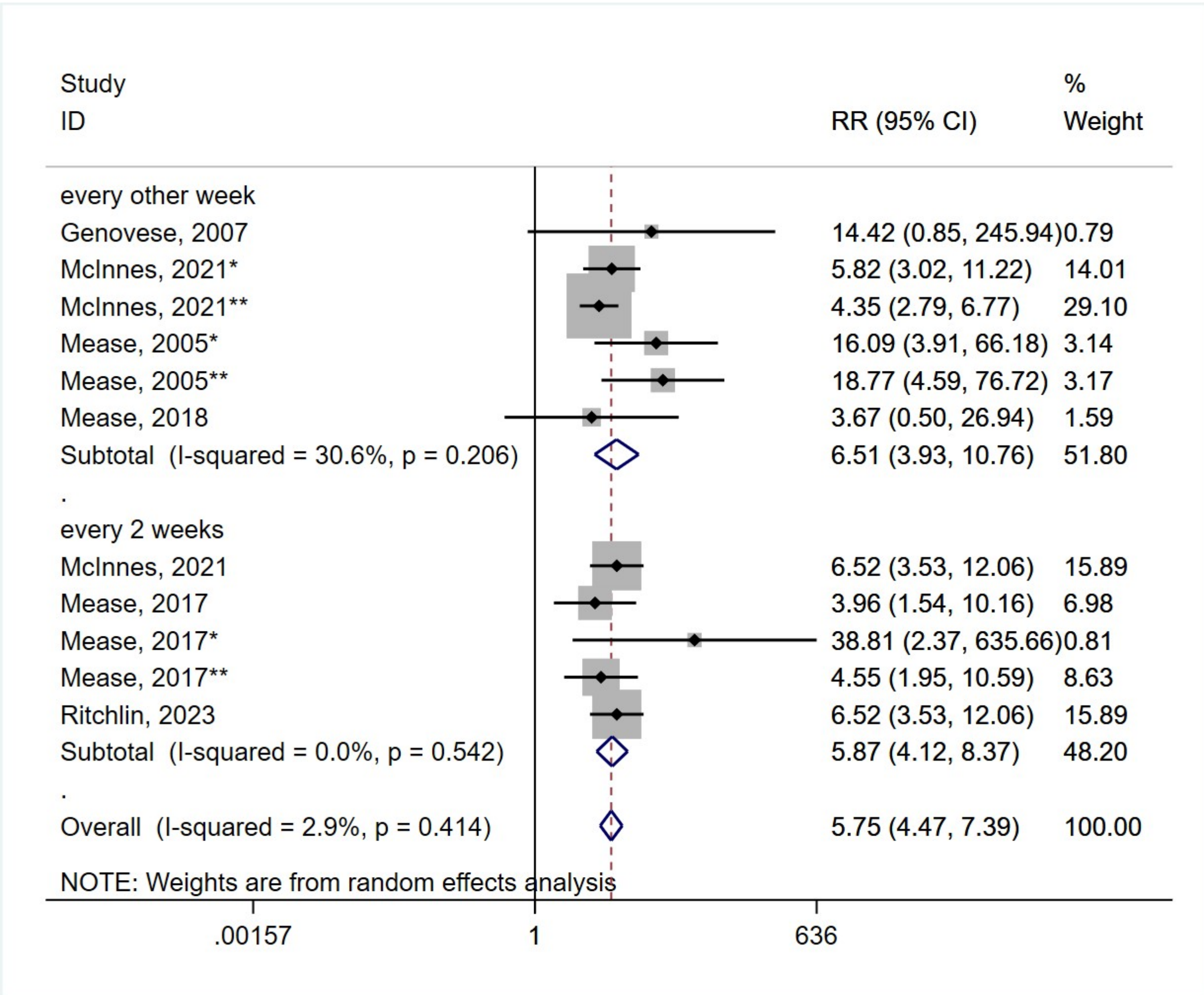
Study
ID
RR (95% CI)
%
Weight
every other week
Genovese, 2007 14.42 (0.85, 245.94) 0.79
McInnes, 2021* 5.82 (3.02, 11.22) 14.01
McInnes, 2021** 4.35 (2.79, 6.77) 29.10
Mease, 2005* 16.09 (3.91, 66.18) 3.14
Mease, 2005** 18.77 (4.59, 76.72) 3.17
Mease, 2018 3.67 (0.50, 26.94) 1.59
Subtotal (I-squared = 30.6%, p = 0.206) 6.51 (3.93, 10.76) 51.80
every 2 weeks
McInnes, 2021 6.52 (3.53, 12.06) 15.89
Mease, 2017 3.96 (1.54, 10.16) 6.98
Mease, 2017* 38.81 (2.37, 635.66) 0.81
Mease, 2017** 4.55 (1.95, 10.59) 8.63
Ritchlin, 2023 6.52 (3.53, 12.06) 15.89
Subtotal (I-squared = 0.0%, p = 0.542) 5.87 (4.12, 8.37) 48.20
Overall (I-squared = 2.9%, p = 0.414) 5.75 (4.47, 7.39) 100.00
NOTE: Weights are from random effects analysis
.00157
1
636
