# Supplementary material for: Efficacy and safety evaluation of adalimumab for psoriatic arthritis: A meta-analysis
Source: Medicine (Baltimore). 2026 Aug 21;105(34):e50295. doi: 10.1097/MD.0000000000050295 (PMC13502872; doi:10.1097/MD.0000000000050295)
Supplement: Supplementary file 21 [file medi-105-e50295-s022.pdf]

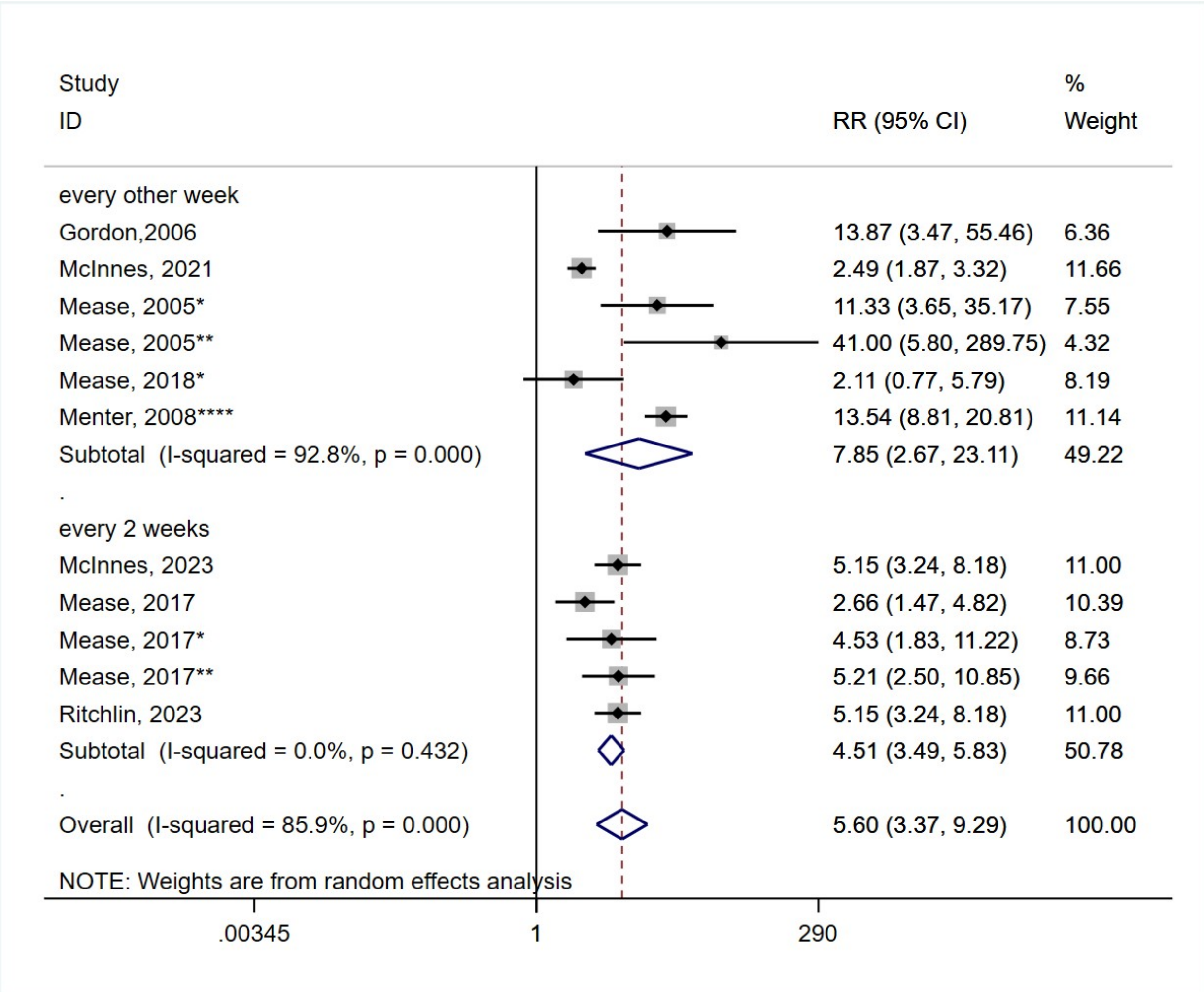
Study
ID
RR (95% CI)
%
Weight
every other week
Gordon,2006
13.87 (3.47, 55.46)
6.36
McInnes, 2021
2.49 (1.87, 3.32)
11.66
Mease, 2005*
11.33 (3.65, 35.17)
7.55
Mease, 2005**
41.00 (5.80, 289.75)
4.32
Mease, 2018*
2.11 (0.77, 5.79)
8.19
Menter, 2008****
13.54 (8.81, 20.81)
11.14
Subtotal (I-squared = 92.8%, p = 0.000)
7.85 (2.67, 23.11)
49.22
every 2 weeks
McInnes, 2023
5.15 (3.24, 8.18)
11.00
Mease, 2017
2.66 (1.47, 4.82)
10.39
Mease, 2017*
4.53 (1.83, 11.22)
8.73
Mease, 2017**
5.21 (2.50, 10.85)
9.66
Ritchlin, 2023
5.15 (3.24, 8.18)
11.00
Subtotal (I-squared = 0.0%, p = 0.432)
4.51 (3.49, 5.83)
50.78
Overall (I-squared = 85.9%, p = 0.000)
5.60 (3.37, 9.29)
100.00
NOTE: Weights are from random effects analysis
.00345
1
290
