# Supplementary material for: Efficacy and safety evaluation of adalimumab for psoriatic arthritis: A meta-analysis
Source: Medicine (Baltimore). 2026 Aug 21;105(34):e50295. doi: 10.1097/MD.0000000000050295 (PMC13502872; doi:10.1097/MD.0000000000050295)
Supplement: Supplementary file 22 [file medi-105-e50295-s023.pdf]

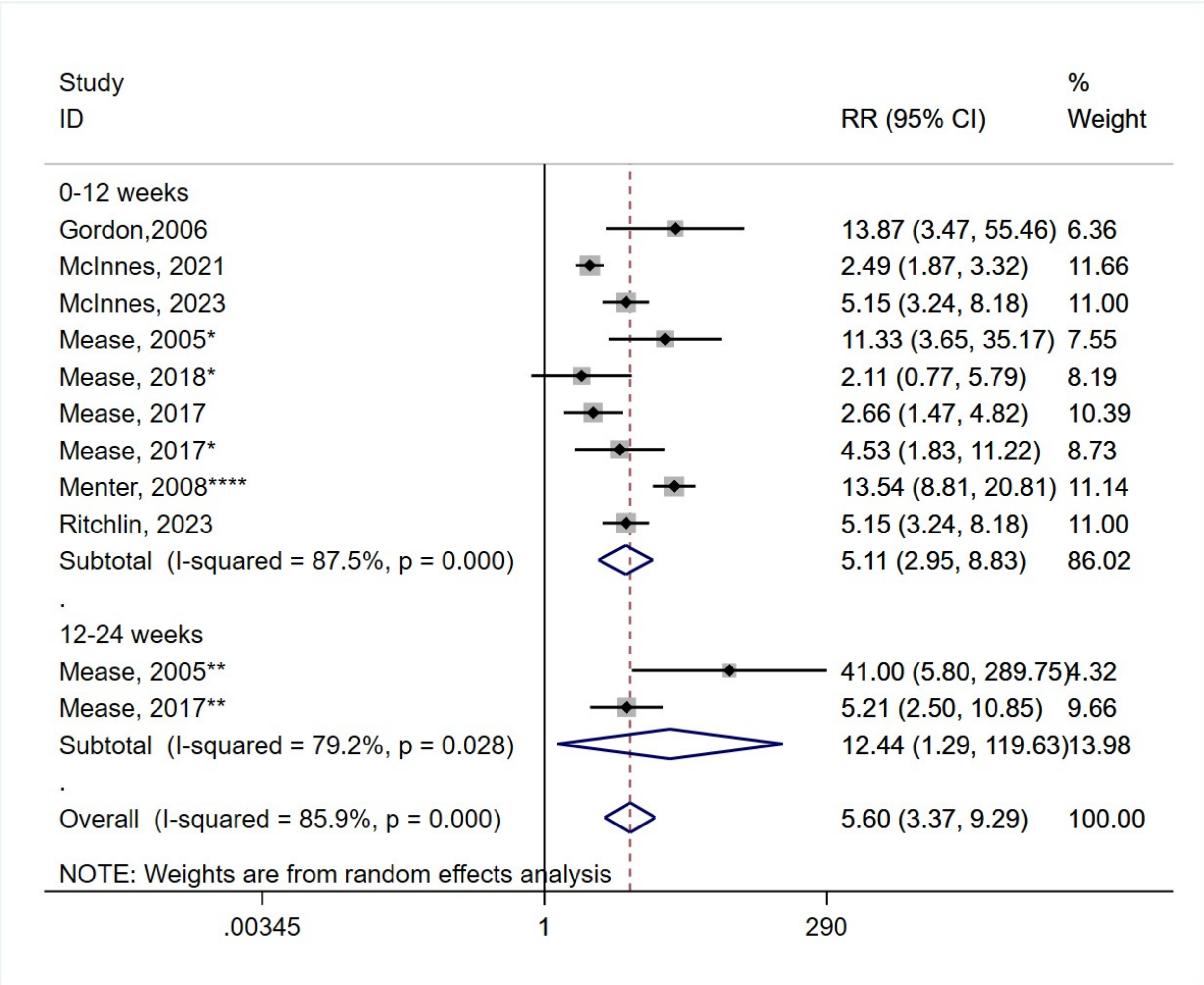

Study
ID
RR (95% CI)
%
Weight
0-12 weeks
Gordon,2006
13.87 (3.47, 55.46) 6.36
McInnes, 2021
2.49 (1.87, 3.32) 11.66
McInnes, 2023
5.15 (3.24, 8.18) 11.00
Mease, 2005*
11.33 (3.65, 35.17) 7.55
Mease, 2018*
2.11 (0.77, 5.79) 8.19
Mease, 2017
2.66 (1.47, 4.82) 10.39
Mease, 2017*
4.53 (1.83, 11.22) 8.73
Menter, 2008****
13.54 (8.81, 20.81) 11.14
Ritchlin, 2023
5.15 (3.24, 8.18) 11.00
Subtotal (I-squared = 87.5%, p = 0.000)
5.11 (2.95, 8.83) 86.02
.
12-24 weeks
Mease, 2005**
41.00 (5.80, 289.75) 4.32
Mease, 2017**
5.21 (2.50, 10.85) 9.66
Subtotal (I-squared = 79.2%, p = 0.028)
12.44 (1.29, 119.63) 13.98
.
Overall (I-squared = 85.9%, p = 0.000)
5.60 (3.37, 9.29) 100.00
NOTE: Weights are from random effects analysis
.00345
1
290
