# Supplementary material for: Efficacy and safety evaluation of adalimumab for psoriatic arthritis: A meta-analysis
Source: Medicine (Baltimore). 2026 Aug 21;105(34):e50295. doi: 10.1097/MD.0000000000050295 (PMC13502872; doi:10.1097/MD.0000000000050295)
Supplement: Supplementary file 23 [file medi-105-e50295-s024.pdf]

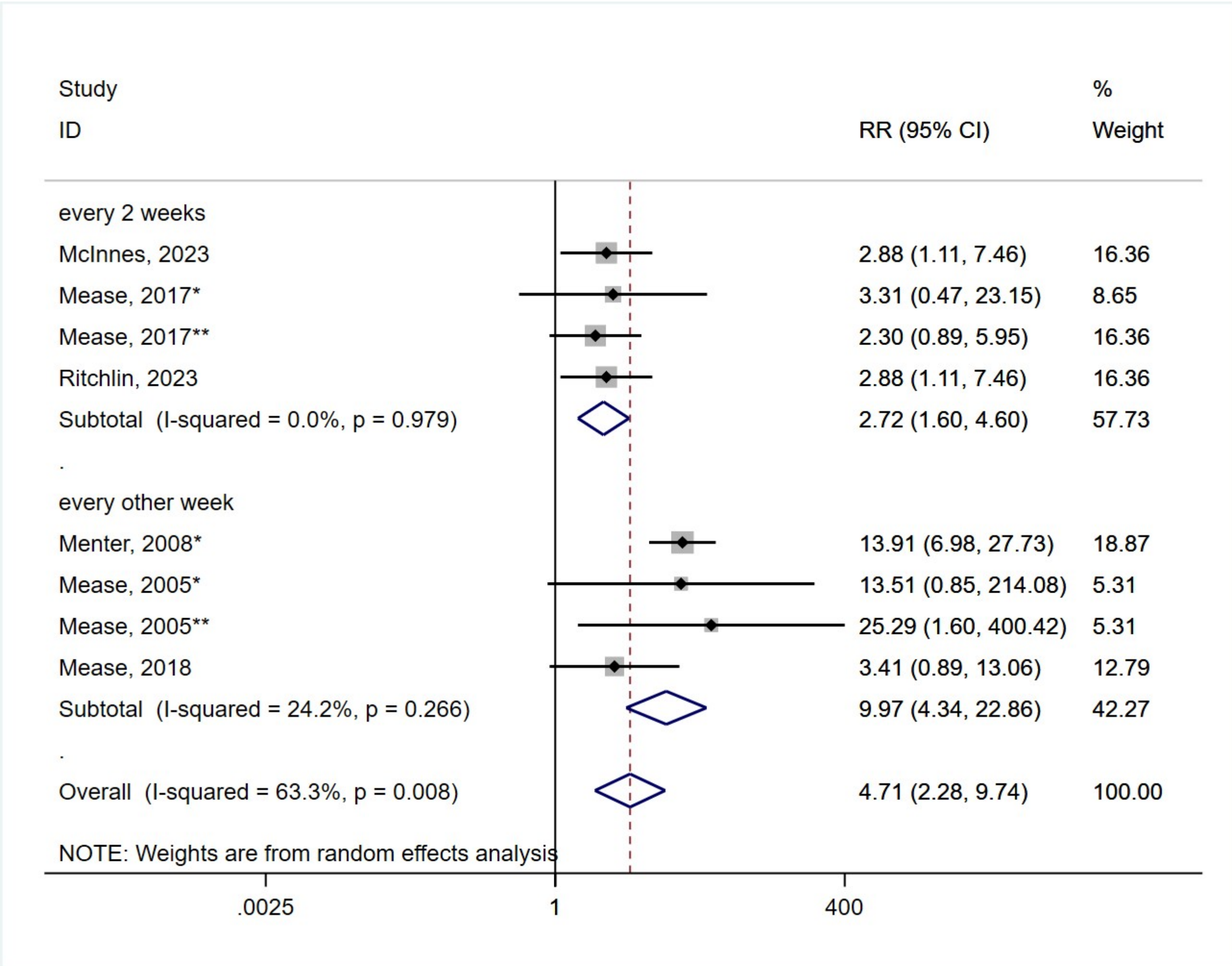
Study
ID
RR (95% CI)
%
Weight
every 2 weeks
McInnes, 2023 2.88 (1.11, 7.46) 16.36
Mease, 2017* 3.31 (0.47, 23.15) 8.65
Mease, 2017** 2.30 (0.89, 5.95) 16.36
Ritchlin, 2023 2.88 (1.11, 7.46) 16.36
Subtotal (I-squared = 0.0%, p = 0.979) 2.72 (1.60, 4.60) 57.73
.
every other week
Menter, 2008* 13.91 (6.98, 27.73) 18.87
Mease, 2005* 13.51 (0.85, 214.08) 5.31
Mease, 2005** 25.29 (1.60, 400.42) 5.31
Mease, 2018 3.41 (0.89, 13.06) 12.79
Subtotal (I-squared = 24.2%, p = 0.266) 9.97 (4.34, 22.86) 42.27
.
Overall (I-squared = 63.3%, p = 0.008) 4.71 (2.28, 9.74) 100.00
NOTE: Weights are from random effects analysis
.0025
1
400
