# Supplementary material for: Efficacy and safety evaluation of adalimumab for psoriatic arthritis: A meta-analysis
Source: Medicine (Baltimore). 2026 Aug 21;105(34):e50295. doi: 10.1097/MD.0000000000050295 (PMC13502872; doi:10.1097/MD.0000000000050295)
Supplement: Supplementary file 25 [file medi-105-e50295-s026.pdf]

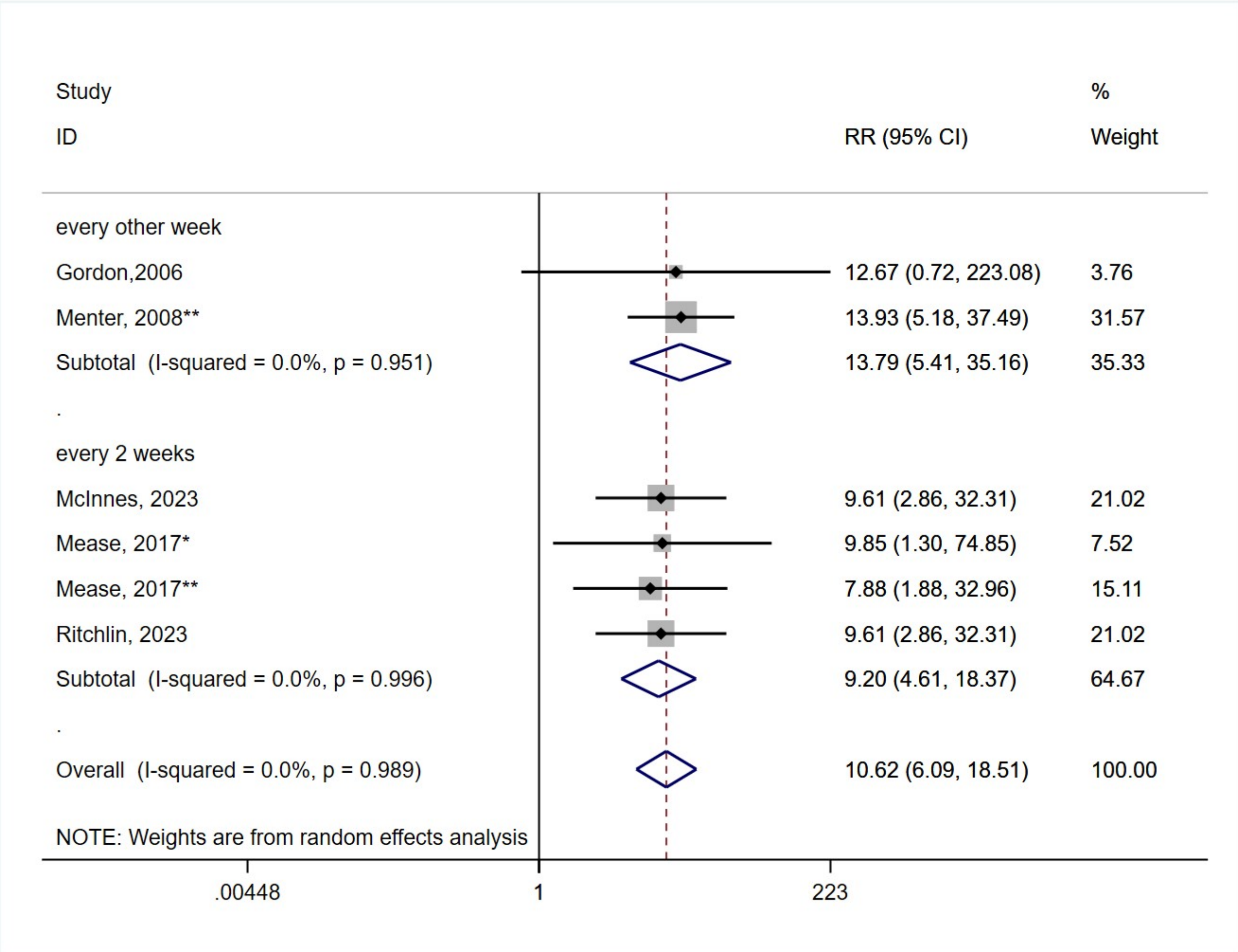

Study
ID
RR (95% CI)
%
Weight
every other week
Gordon,2006
12.67 (0.72, 223.08)
3.76
Menter, 2008**
13.93 (5.18, 37.49)
31.57
Subtotal (I-squared = 0.0%, p = 0.951)
13.79 (5.41, 35.16)
35.33
.
every 2 weeks
McInnes, 2023
9.61 (2.86, 32.31)
21.02
Mease, 2017*
9.85 (1.30, 74.85)
7.52
Mease, 2017**
7.88 (1.88, 32.96)
15.11
Ritchlin, 2023
9.61 (2.86, 32.31)
21.02
Subtotal (I-squared = 0.0%, p = 0.996)
9.20 (4.61, 18.37)
64.67
.
Overall (I-squared = 0.0%, p = 0.989)
10.62 (6.09, 18.51)
100.00
NOTE: Weights are from random effects analysis
.00448
1
223
