# Supplementary material for: Efficacy and safety evaluation of adalimumab for psoriatic arthritis: A meta-analysis
Source: Medicine (Baltimore). 2026 Aug 21;105(34):e50295. doi: 10.1097/MD.0000000000050295 (PMC13502872; doi:10.1097/MD.0000000000050295)
Supplement: Supplementary file 26 [file medi-105-e50295-s027.pdf]

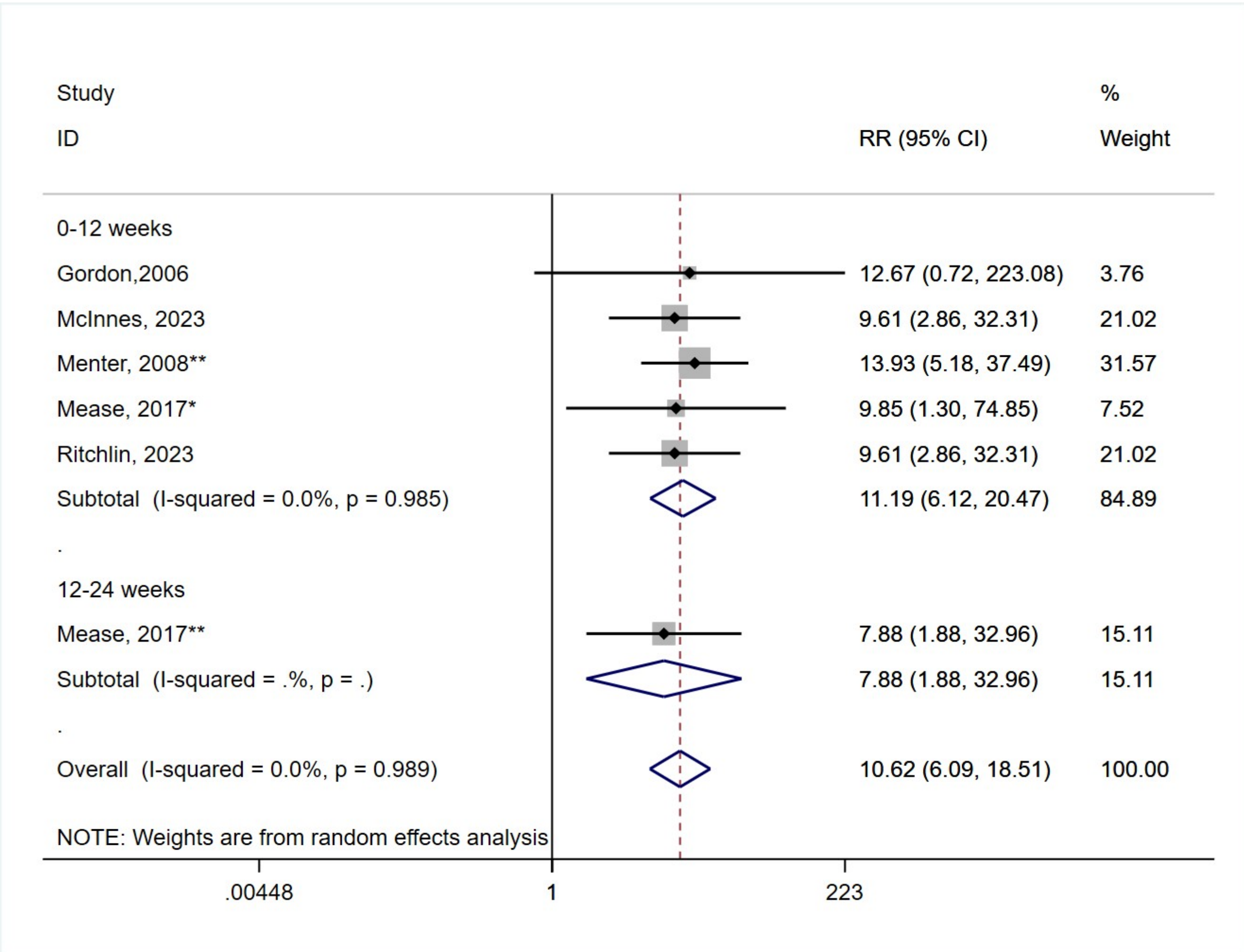
Study
ID
RR (95% CI)
%
Weight
0-12 weeks
Gordon,2006
12.67 (0.72, 223.08)
3.76
McInnes, 2023
9.61 (2.86, 32.31)
21.02
Menter, 2008**
13.93 (5.18, 37.49)
31.57
Mease, 2017*
9.85 (1.30, 74.85)
7.52
Ritchlin, 2023
9.61 (2.86, 32.31)
21.02
Subtotal (I-squared = 0.0%, p = 0.985)
11.19 (6.12, 20.47)
84.89
.
12-24 weeks
Mease, 2017**
7.88 (1.88, 32.96)
15.11
Subtotal (I-squared = .%, p = .)
7.88 (1.88, 32.96)
15.11
.
Overall (I-squared = 0.0%, p = 0.989)
10.62 (6.09, 18.51)
100.00
NOTE: Weights are from random effects analysis
.00448
1
223
