# Supplementary material for: Efficacy and safety evaluation of adalimumab for psoriatic arthritis: A meta-analysis
Source: Medicine (Baltimore). 2026 Aug 21;105(34):e50295. doi: 10.1097/MD.0000000000050295 (PMC13502872; doi:10.1097/MD.0000000000050295)
Supplement: Supplementary file 27 [file medi-105-e50295-s002.docx]

**Table S1.** Detailed information table of the included studies

| Author, year | ClinicalTrials.gov registration number | Phase | Treatment regimen and dose | | Trial enrollment size | | Treatment plan  (Research therapy) | Primary outcome measures |
| --- | --- | --- | --- | --- | --- | --- | --- | --- |
|  |  |  | Treatment | Control | Treatment | Control |  |  |
| Mease, 2005 | Not Mentioned | Not Mentioned | ADA,40mg,  every other week,  subcutaneously | PBO,  every other week,  subcutaneously | 151 | 162 | ADA,PBO | ACR20,ACR50,ACR70,PASI75,  PASI90,PsARC |
| Gordon, 2006 | Not Mentioned | Not Mentioned | ADA,40mg,80mg,  every other week,weekly,  subcutaneously | PBO,  weekly,  subcutaneously | 45,50 | 52 | ADA,PBO | PASI75,PASI100 |
| Genovese, 2007 | Not Mentioned | Ⅲ | ADA,40mg,  every other week,  subcutaneously | PBO,  subcutaneously | 51 | 49 | ADA,PBO | ACR20,ACR50,ACR70,PsARC |
| Menter, 2008 | NCT00237887 | Ⅲ | ADA,40mg,80mg,  every other week,  subcutaneously | PBO,  subcutaneously | 814 | 398 | ADA,PBO | PASI75,PASI90,PASI100 |
| Revicki, 2008 | Not Mentioned | Not Mentioned | ADA,40mg,80mg,  PBO capsules,  weekly,  subcutaneously | PBO,  weekly,  oral, subcutaneously | 103 | 53 | ADA,PBO,MTX |  |
| van Kuijk AW, 2009 | Not Mentioned | Not Mentioned | ADA,40mg,  every other week,  subcutaneously | PBO,  subcutaneously | 12 | 12 | ADA,PBO |  |
| Paramarta, 2013 | Not Mentioned | Not Mentioned | ADA,40mg,  every other week,  subcutaneously | PBO,  subcutaneously | 20 | 20 | ADA,PBO |  |
| Mease, 2015 | NCT01064856 | Ⅲ | ADA,40mg,  every other week,  subcutaneously | PBO,  subcutaneously | 84 | 81 | ADA,PBO |  |
| Mease, 2017 | NCT01695239 | Ⅲ | ADA,40mg,  every 2 weeks,  subcutaneously | PBO,  subcutaneously | 101 | 106 | ADA,PBO,Ixekizumab | ACR20,ACR50,ACR70,PASI75,PASI90,  PASI100 |
| Mease, 2017 | NCT01877668 | Ⅲ | ADA,40mg,  every 2 weeks,  subcutaneously | PBO,  subcutaneously | 106 | 105 | ADA,PBO,Tofacitinib | ACR20,ACR50,ACR70,PASI75,MDA |
| Mease, 2018 | NCT02349451 | Ⅱ | ADA,40mg,  every other week,  subcutaneously | PBO,weekly,  subcutaneously | 72 | 24 | ADA,PBO,ABT-122 | ACR20,ACR50,ACR70,PASI75,  PASI90 |
| Gottlieb, 2018 | NCT01695239 | Ⅲ | ADA,40mg,every 2 weeks, subcutaneously | PBO,  subcutaneously | 101 | 106 | ADA,PBO,Ixekizumab |  |
| Strand, 2019 | NCT01877668 | Ⅲ | ADA,40mg,  every 2 weeks,  subcutaneously | PBO,  subcutaneously | 106 | 105 | ADA,PBO,Tofacitinib |  |
| McInnes, 2021 | NCT03104400 | Ⅲ | ADA,40mg,  every other week,  subcutaneously | PBO,  every other week,  subcutaneously | 449 | 423 | ADA,PBO,Upadacitinib | ACR20,ACR50,ACR70,PASI75,  Complete resolution of enthesitis,MDA |
| Strand, 2021 | NCT03104400 | Ⅲ | ADA,40mg,  every other week,  subcutaneously | PBO,  every other week,  subcutaneously | 429 | 423 | ADA,PBO,Upadacitinib |  |
| McInnes, 2023 | NCT03895203 | Ⅲ | ADA,40mg,  every 2 weeks,  subcutaneously | PBO,  every 2 weeks,  subcutaneously | 140 | 281 | ADA,PBO,Bimekizumab | ACR20,ACR50,ACR70,PASI75,PASI90,  PASI100,Complete resolution of enthesitis,  MDA |
| Ritchlin, 2023 | NCT03895203 | Ⅲ | ADA,40mg,  every 2 weeks,  subcutaneously | PBO,  every 2 weeks,  subcutaneously | 140 | 281 | ADA,PBO,Bimekizumab | ACR20,ACR50,ACR70,PASI75,PASI90,  PASI100,Complete resolution of enthesitis,  MDA |

ADA: Adalimumab; PBO: Placebo; PsARC: Psoriatic arthritis response criteria; MDA: Minimal disease activity
